# Structural and Functional Characterization of New *Sso*Pox Variant Points to the Dimer Interface as a Driver for the Increase in Promiscuous Paraoxonase Activity

**DOI:** 10.3390/ijms21051683

**Published:** 2020-03-01

**Authors:** Yoko Suzumoto, Orly Dym, Giovanni N. Roviello, Franz Worek, Joel L. Sussman, Giuseppe Manco

**Affiliations:** 1 Institute of Biochemistry and Cell Biology, CNR, 80131 Naples, Italy; yoko.suzumoto@biogem.it; 2 The Weizmann Institute of Science, Rehovot, 76100 Israel; Orly.Dym@weizmann.ac.il (O.D.); joel.sussman@weizmann.ac.il (J.L.S.); 3 Institute of Biostructures and Bioimages, CNR, 80131 Naples, Italy; giroviel@unina.it; 4 Bundeswehr Institute of Pharmacology and Toxicology, Munich, Germany; franzworek@bundeswehr.org

**Keywords:** Molecular evolution, Phosphotriesterase-Like-Lactonases (PLLs), organophosphate (OP) compounds, nerve agents, enzymatic detoxification/remediation

## Abstract

Increasing attention is more and more directed toward the thermostable Phosphotriesterase-Like-Lactonase (PLL) family of enzymes, for the efficient and reliable decontamination of toxic nerve agents. In the present study, the DNA Staggered Extension Process (StEP) technique was utilized to obtain new variants of PLL enzymes. Divergent homologous genes encoding PLL enzymes were utilized as templates for gene recombination and yielded a new variant of SsoPox from *Saccharolobus solfataricus*. The new mutant, V82L/C258L/I261F/W263A (4Mut) exhibited catalytic efficiency of 1.6 × 10^5^ M^−1^ s^−1^ against paraoxon hydrolysis at 70°C, which is more than 3.5-fold and 42-fold improved in comparison with C258L/I261F/W263A (3Mut) and wild type SsoPox, respectively. 4Mut was also tested with chemical warfare nerve agents including tabun, sarin, soman, cyclosarin and VX. In particular, 4Mut showed about 10-fold enhancement in the hydrolysis of tabun and soman with respect to 3Mut. The crystal structure of 4Mut has been solved at the resolution of 2.8 Å. We propose that, reorganization of dimer conformation that led to increased central groove volume and dimer flexibility could be the major determinant for the improvement in hydrolytic activity in the 4Mut.

## 1. Introduction

The ever-increasing number of organophosphate (OP) pesticides poisoning, as well as intensive use of OP chemical warfare agents (CWA) in civil war and terrorist acts have urged the immediate establishment of sustainable and reliable methods for sensing, decontamination and detoxification of OP compounds [1,2]. Enzymatic detoxification has attracted an increasing attention as an alternative to conventional OP-detoxification methods, such as chemical treatment, incineration, bleaching and volatilization [3,4,5,6,7,8,9,10,11]. One of the most studied OP-degrading enzymes includes phosphotriesterase from mesophilic bacteria, *Brevundimonas diminuta* MG (*bd*PTE) [12]. *bd*PTE has exhibited hydrolytic activities against a wide range of OP insecticides such as ethyl-paraoxon, parathion, methyl parathion, dursban, coumaphos, diazinon, fensulfothion, and cyanophos. The catalytic efficiency (*k*_cat_/K_M_) towards paraoxon is particularly high, reaching at 4.0 × 10^7^ M^−1^s^−1^ [13,14,15,16]. In addition, *bd*PTE demonstrated the ability to degrade both G-series (sarin, soman, tabun, cyclosarin) and V-series (VX and VR) nerve gases [13,14,15,16]. The hydrolytic activities against OP compounds demonstrated by *bd*PTE have shown a potential of these enzymes to apply for practical decontamination of toxic OP compounds. On the other hand, it is inevitable that OP-degrading enzymes must possess the stability under wide range of temperatures, pH, and the presence of organic solvents, in consideration of the real decontamination conditions of OP-contaminated soil, surfaces, air, water and waste.

In this context, our group has focused on the studies of an enzyme family of lactonases with promiscuous phosphotriesterase activity, dubbed Phosphotriesterase-Like-Lactonases (PLLs) [17,18,19,20,21,22]. PLL family enzymes are closely related to mesophilic *bd*PTEs, being classified in the same amidohydrolase superfamily. Several thermostable PLL enzymes have been identified from either hyperthermophilic archaea or thermophilic/extremophilic bacteria [23,24,25,26,27]. Their characteristic thermal stability made PLL enzymes one of the ideal candidates to apply for OP-degradation in practical decontamination issues. Therefore, an important study on PLL enzymes is the enhancement of promiscuous phosphotriesterase activity without compromising their exceptional thermal stability.

A variety of both rational and directed evolution approaches have been applied on thermostable PLL enzymes and yielded engineered PLL variants. For example, hydrolytic activity of DrOPH from *Deinococcus radiodurans* was improved toward two OP pesticides, ethyl and methyl paraoxon, through both structure-based and random mutagenesis approaches [23]. More recently, Hoque and coworkers generated an engineered variant of GKL/GKaP from *Geobacillus kaustophilus* by stepwise loop insertion strategy (StLois), in which randomized residue pairs were inserted in loop 7 of GKL in a stepwise manner. Obtained new variant, ML7-B6, has shown a catalytic efficiency of 2.4 × 10^5^ M^−1^ s^−1^ at 65 °C against paraoxon [28].

Previously our group reported the characterization of *Sso*Pox variants generated by directed evolution and semi-rational design [19,22]. In addition, W263 residue in the active site loop 8 was shown to play a critical role on substrate specificity through modulating the flexibility of loop 8. The substitution of W263 with all other amino acids resulted in enhanced paraoxonase activity [19,29]. Recently, we reported that engineered *Sso*Pox triple mutant, C258L/I261F/W263A (3Mut), has shown catalytic efficiency (*k*_cat_/K_M_) for paraoxonase activity approaching 4.5 ± 0.8 × 10^4^ M^−1^ s^−1^ at 65 °C. We speculated that the improvement in 3Mut is presumably due to the combination of the enlargement of active site by the alanine substitution at position 263, accompanied by the hydrophobic patch created by both leucine and phenylalanine at positions 258 and 261, respectively. These three positions are among the 16 amino acid residues identified through a structural alignment between *Sso*Pox and *bd*PTE, which revealed the involvement of these 16 residues in the lactone substrate binding site. Furthermore, we performed site-saturated mutagenesis by means of smart library construction on 6 additional residues (99, 229, 255, 257, 265, 266) that were identified through a bioinformatics approach. However, we did not obtain variants with better paraoxonase activity than 3Mut [22]. Later, another group also reported the detailed characterization on *Sso*Pox 3Mut (αsA1) and other variants showing enhanced phosphotriesterase activity [30]. It was demonstrated that these variants showed enlargement of active site with respect to wt *Sso*Pox, confirming our hypothesis obtained from the model analysis [22,30].

In the present study, starting from 3Mut, we have designed a new directed evolutionary approach based on DNA StEP gene recombination method for further improvement of promiscuous phosphotriesterase activity of *Sso*Pox. The obtained new mutant, V82L/C258L/I261F/W263A (4Mut), exhibited catalytic efficiency at 1.6 × 10^5^ M^−1^ s^−1^ against paraoxon hydrolysis at 70 °C.

We further performed in-depth structural analyses by solving 3D structure, computational as well as circular dichroism (CD) analyses in search for the structural determinants for the improved catalytic activity in 4Mut. Here we demonstrate that a single mutation not located at the active site can alter the dimer flexibility through reorganization of dimer conformation. The increased dimer flexibility could further bring about the improvement in hydrolytic activity. This hypothesis can be also applicable for molecular evolution of this class of enzymes.

## 2. Results

### 2.1. Directed Evolution for the Generation of New Mutant PLL

DNA StEP recombination method generally requires more than one kind of homologous genes as templates for the syntheses of polynucleotides [31]. During gene recombination, growing DNA fragments are subject to anneal to one of the templates due to the sequence complementarity. At each cycle consisting of denaturation and extremely abbreviated annealing/extension reaction, the template is switched so that the resulting DNA fragments carry sequence information from different parental genes [31].

At the first stage of the generation of PLL mutant library, we utilized 6 PLL genes, including *Sso*Pox triple mutant *C258L/I261F/W263A (3Mut)*, *sislac* from *Sulfolobus islandicus*, *sacpox* from *Sulfolobus acidocaldarius*, *vmolac* from *Vulcanisaeta moutnovskia*, *gkl* from *Geobacillus kaustophilus*, and *dr0930* from *Deinococcus radiodurans* as template genes for recombination. Sequence alignments of DNA and encoded proteins from these 6 genes are shown in Figures S1 and S2, respectively. Tables S1 and S2 summarize the sequence identities between each 6 genes.

Prior to screening for variants with improved paraoxonase activity, esterase activity assay was carried out as primary screening. In situ esterase activity assay for *E. coli* BL21 (DE3) expressing mutant PLL enzymes was established as shown in Figure S2. As previously reported, esterase activity is one of the promiscuous activities known for PLL enzymes [18,20], therefore utilized as primary screening for new mutant library in this study. Esterase activity of each mutant enzyme was simply detected by intensity of violet color on the nitrocellulose membrane (Figure S3). The colony expressing mutant enzyme with higher esterase activity shows stronger violet color. Through primary esterase activity assay, inactive mutant PLL enzymes were avoided for further paraoxonase activity assay. In fact, we assumed that many of mutant PLL enzymes would be inactive presumably due to the folding problems caused by randomly introduced mutations. In other words, only active mutant PLL enzymes have been selected through esterase activity assay.

More than 20,000 colonies were analyzed by primary esterase activity assay. Among them, colonies showing high esterase activity were selected for secondary screening for phosphotriesterase activity assay in 96-well plates using paraoxon as substrate. Paraoxonase activity of these variants was compared with that in 3Mut. Approximately 2000 variants were screened by paraoxonase activity assay, however, we were not able to identify variants showing higher paraoxonase activity than that of 3Mut.

For the verification of gene recombination in this study, we selected four mutants (B1, B9, C2 and D5) for further investigation. The comparison of paraoxonase activity between these 4 variants and 3Mut is shown in Figure S4. It was revealed that after incubation at 70 °C for 30 min, paraoxonase activities of these 4 variants were less than 40% with respect to 3Mut. We also performed complete sequencing of these 4 variants and sequence alignment with template genes. As shown in Figure S5, none of variants were identical to any of template genes used for our DNA StEP method, indicating that homologous PLL genes from different species were successfully recombined through DNA StEP approach. However, it should be noted that, the sequence identities among 4 variants were high, approaching more than 94.0%. In addition, these 4 variants possessed high sequence identities with either SisLac or 3Mut (Table S3). Therefore, it is likely that gene recombination rate in these selected variants were quite low. Although it is not clear if randomly recombined genes were eliminated through primary esterase activity screening, we rearranged our DNA StEP strategy in search for variants with increased paraoxonase activities.

**Figure 1:**
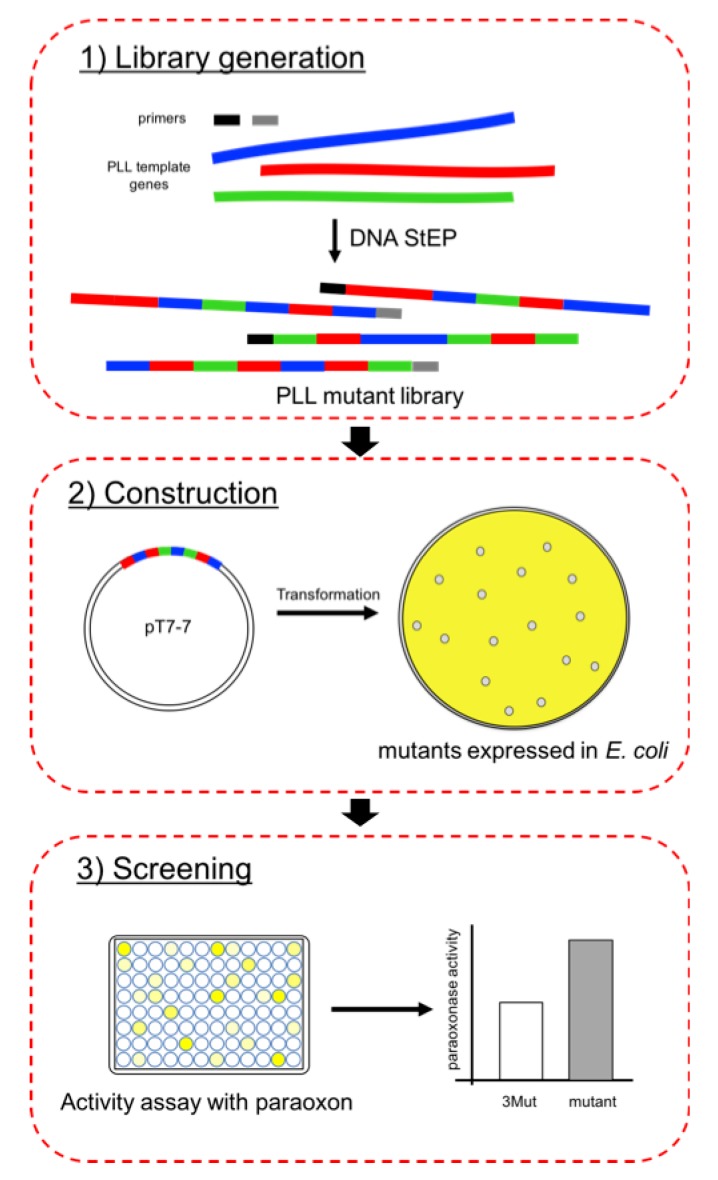
Schematic view of the directed evolution approach used in this work.

In the next DNA StEP gene recombination experiment (Figure 1), we selected two PLL genes, *dr0930* and *gkl*, as templates to recombine with *Sso*Pox triple mutant *C258L/I261F/W263A* for the generation of new variants. These two bacterial PLL genes show low sequence identities with respect to *C258L/I261F/W263A.* GKL shows 46.7% identities at DNA level, and 35.8% at protein level. *Dr*0930 exhibits even lower sequence identities against 3Mut, which were 42.6% and 29.9% at DNA and protein level, respectively (Tables S1 and S2). It was expected that utilizing homologous PLL genes with low sequence identities could result in the generation of new variants in two different ways; one could be obtaining variants with high divergence, by building up whole sequence of variants utilizing randomly selected piece of sequence information from divergent homologous genes. On the other hand, we could not eliminate the possibility in which low sequence identities between templates could result in unfavorable template switching. As a result, DNA fragments could be generated utilizing only one gene as template. In the latter case, even though the recombination rate between templates could be quite low, it is still attractive since only one or a few mutations resulted in improved enzymatic activity in previous mutagenesis studies of PLL enzymes [19,22] unless these mutants are identical with template genes.

We have screened up to 200 variants for paraoxonase activity and identified one mutant showing approximately 1.5-fold improved paraoxonase activity compared with that of 3Mut. Sequence analysis revealed that this new mutant contains the single substitution from valine to leucine at position 82, with respect to 3Mut. This single mutation was obtained through a single nucleotide change from GTG to TTG.

### 2.2. Biochemical Characterization of 4Mut Mutant

#### 2.2.1. Optimal Metal Ion

**Figure 2:**
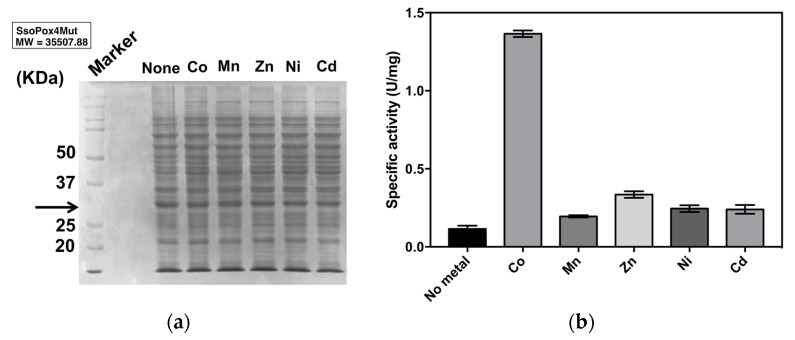
Metal ions analysis for the enhancement of paraoxonase activity of *Sso*Pox 4Mut. (**a**) SDS-PAGE of crude extracts. The band derived from the expression of 4Mut is indicated by the arrow. (**b**) Paraoxonase activity of 4Mut without metal or in the presence of different kind of metal including cobalt, manganese, zinc, nickel and cadmium. Assays were performed using crude extracts.

Several reports suggest that catalytic activity of PLL enzymes as well as PTE depends on the presence of metal ions in the active site. In addition, the species of metals also modulate the activity [18,20,21,24,25,26]. In order to examine the metal ions for the enhancement of paraoxonase activity, new mutant V82L/C258L/I261F/W263A (4Mut) was expressed in *E. coli* BL21(DE3) under the presence of different metal ions including Co^2+^, Mn^2+^, Zn^2+^, Ni^2+^ or Cd^2+^. The effect of different metals in the growth medium on the expression level of 4Mut was analyzed by SDS-PAGE. By observing the signals at around 35 KDa, which corresponds to the molecular mass of 4Mut monomer, it was confirmed that there was no significant difference in the expression level of 4Mut in the absence or presence of different metal ions (Figure 2a). The paraoxonase activity of 4Mut was enhanced more than 10-fold in the presence of Co^2+^ compared with the crude extract without metal ions (Figure 2b). For other metals, paraoxonase activities were enhanced 2- to 3-fold with respect to the crude extract without any metals. These results suggest that cobalt enhances the paraoxonase activity of 4Mut most efficiently, in agreement with *Sso*Pox and its mutants reported previously [18,19,22].

For further characterization, 4Mut was expressed in *E. coli* BL21(DE3) at a medium scale (1.5 L) and purified under the presence of 0.5 mM CoCl_2_ in the buffer solution throughout the purification process. In order to exclude any influence on enzymatic characterization caused by contaminating proteins, we only choose the fraction containing 4Mut with more than 95% purity after size-exclusion chromatography as the final step of the purification. 0.6 mg of highly pure (> 95%) 4Mut was obtained from 1.5 L cell culture and utilized for the enzymatic characterization.

#### 2.2.2. pH Optima

**Figure 3:**
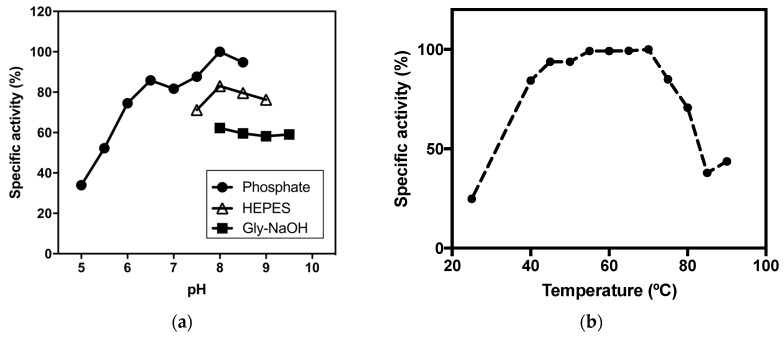

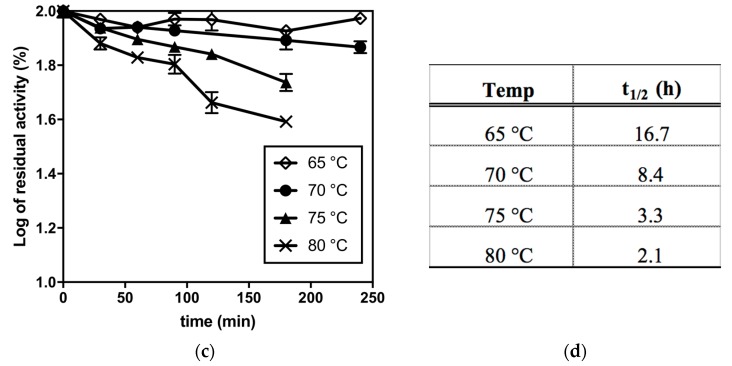
(**a**) pH dependency of paraoxonase activity of 4Mut. Paraoxonase activity of 4Mut was measured at different pH values ranging from 5.0 to 9.5 by using phosphate buffer (pH 5.0–8.5), HEPES-NaOH (pH 7.5–9.0) and Gly-NaOH (pH8.0–9.5). (**b**) Thermophilicity curve of paraoxonase activity of 4Mut. (**c**) Thermal stability of 4Mut. The enzyme (1 mg/mL) in 20 mM HEPES pH 8.5, 0.5 mM CoCl_2_, 200 mM NaCl was incubated at either 65 °C (squares), 70 °C (full circles), 75 °C (full triangles), or 80 °C (cross). Samples were withdrawn at indicated times and analyzed by standard assay at 70 °C. Logarithm of residual activity (%) was calculated against a non-incubated sample. Half lives (t_1/2_) at each temperature are shown in (**d**).

The pH dependency of 4Mut on paraoxonase activity was examined at 70 °C. In order to cover a wide range of pH from 5.0 to 9.5, three different kinds of buffers (Na_2_HPO_4_/NaH_2_PO_4_, HEPES-NaOH and Gly-NaOH) were utilized. The absorbance was measured at 348 nm, the pH-independent isosbestic point between p-nitrophenol and p-nitrophenolate ion. As shown in Figure 3a, the highest paraoxonase activity of 4Mut was observed at pH 8.0 at 70 °C. This pH is slightly lower than the value reported for 3Mut at pH 8.5 at 65 °C, but is similar to *Sso*Pox and its single mutant [18,19,22]. At the alkaline pH range higher than 8.0, a slight decrease in the paraoxonase activity was observed. In contrast, the hydrolytic activity of 4Mut was considerably decreased near the acidic pH.

#### 2.2.3. Thermophilicity

The thermophilicity curve of paraoxonase activity of 4Mut was obtained at 25 °C and in temperatures ranging from 40 °C to 85 °C at pH 8.0 (Figure 3b). It was revealed that thermophilicity of 4Mut showed a different trend from that reported for 3Mut. In fact, 4Mut did not show clear optimal temperature, but it maintained high activity in the temperature range between 55 °C and 70 °C, while 3Mut exhibited clear optimal temperature at 65 °C [22]. Even though the activity at 25 °C was approximately 25 % of that at 70 °C, the paraoxonase activity of 4Mut was maintained more than 80% at 40 °C with respect to the value obtained at 70 °C. On the other hand, at the temperatures higher than 70 °C, the catalytic activity was decreased sharply. The activity at 85 °C was less than 40 % compared with the value obtained at 70 °C (Figure 3b).

#### 2.2.4. Thermal stability of 4Mut

We assessed the thermal stability of 4Mut by measuring paraoxonase activities after incubating the enzyme for certain amount of time up to 4 h at different temperatures (65, 70, 75 and 80 °C). Figure 3c shows the thermal stability analyses on 4Mut. The half life (t_1/2_) at each temperature showed that 4Mut retains half of its paraoxonase activity for 16.7 h at 65 °C, and 8.4 h at 70 °C (Figure 3d). At 75 and 80 °C, the t_1/2_ values were determined as 3.3 h and 2.1 h, respectively. These values obtained for 4Mut indicate that 4Mut possesses a decreased thermal stability compared with 3Mut, which showed no activity changes at 65 °C and 75 °C. On the other hand, determined thermal stability of 4Mut was higher than those observed for W263F (t_1/2_ = 200 min at 70 °C, and 90 min at 80 °C, respectively) [22].

### 2.3. Enzymatic Characterization of 4Mut

#### 2.3.1. Activity on Phosphotriesters

Even though PLL enzymes exhibit significant phosphotriesterase activities particularly on paraoxon, they are natural lactonases that catalyze the hydrolysis of lactones and acyl-homoserine lactones (AHLs) as their main function [17]. In addition, promiscuous carboxylesterase activities have been detected in this class of enzymes [18,20]. Thus, we examined purified 4Mut for hydrolytic activities on phosphotriesters, carboxylesters and lactones. Table 1 summarizes the substrate profile of 4Mut. The enzyme displays phosphotriesterase activities on paraoxon, methyl-paraoxon and methlyl-parathion as observed in *Sso*Pox wild-type [18]. In particular, specific activities on paraoxon and methyl-paraoxon were remarkably high. Therefore, we further performed the kinetic analysis on phophotriesterase activities of 4Mut against these two substrates at its optimal condition as experimentally determined above (pH 8.0, at 70 °C). Table 2 shows the kinetic data of phophotriesterase activity of 4Mut against paraoxon and methyl-paraoxon with comparison to *Sso*Pox and its mutants. The specific activity of 4Mut against paraoxon reached 206.4 ± 18.3 U/mg at 70 °C. This value is about 500-fold improved compared with *Sso*Pox [18]. With respect to W263F and 3Mut, 16-fold and 1.6-fold increases were observed, respectively [19,22]. In terms of *k*_cat_ and K_M_ values, 4Mut showed 2-fold improvement in both parameters with respect to 3Mut. As a result, catalytic efficiency (*k*_cat_/K_M_) reached 1.6 × 10^5^ M^−1^ s^−1^, which is more than 3.5-fold and 42-fold improved in comparison with 3Mut and *Sso*Pox, respectively.

On the other hand, hydrolytic activity of 4Mut on methyl-paraoxon (50.9 ± 12.0 U/mg) was slightly decreased in comparison with that of 3Mut obtained at pH 8.5, 65 °C. The *k*_cat_ and K_M_ values of 4Mut for the hydrolysis of methyl-paraoxon were determined as 30.1 s^−1^ and 0.871 mM, respectively, resulting in *k*_cat_/K_M_ with 3.5 × 10^4^ M^−1^ s^−1^.

Kinetic parameters for hydrolytic activity of 4Mut against paraoxon were also examined at 25 °C (Table 2). The determined values of *k*_cat_, K_M_ and *k*_cat_/K_M_ for the paraoxonase activity of 4Mut at 25 °C were 33.0 s^−1^, 0.645 mM and 5.1 × 10^4^ M^−1^ s^−1^, respectively. The catalytic efficiency was improved about 2.8-fold compared with the value obtained for 3Mut at 25 °C [22].

#### 2.3.2. Effects of SDS on Paraoxonase Activity of 4Mut

**Figure 4:**
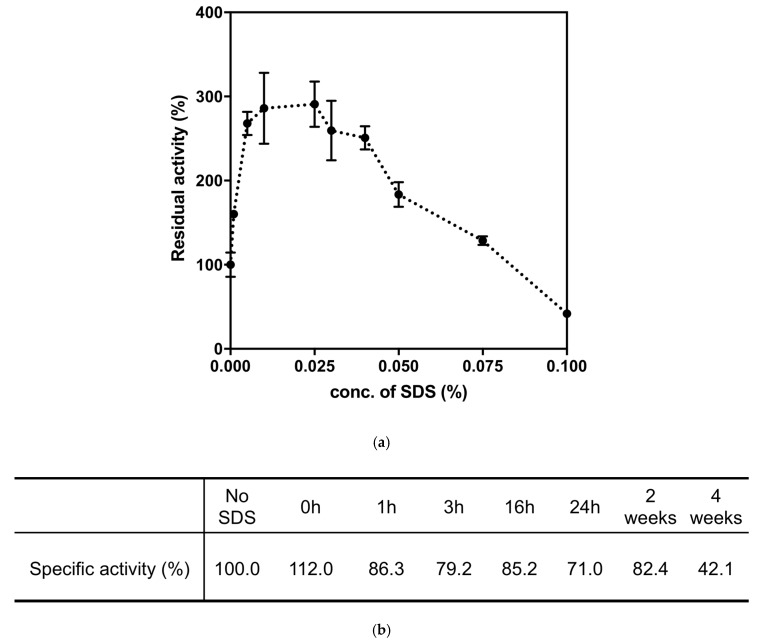
(**a**) Effect of SDS on paraoxonase activity of 4Mut. Standard assay was performed under the presence of different concentration of SDS as indicated. (**b**) Stability of 4Mut on SDS. 4Mut was incubated in buffer (20 mM HEPES-NaOH pH 8.5, 0.5 mM CoCl_2_, 200 mM NaCl) with 0.025% SDS at room temperature. Samples were withdrawn at indicated times and analyzed by standard assay at 70 °C.

Previously it was reported that promiscuous phosphotriesterase activity of *Sso*Pox and its mutants have been strongly enhanced by anionic detergents such as SDS and DOC [19,22,32]. The hydrolytic activities of *Sso*Pox and 3Mut increased 17-fold (at 70 °C) and 2-fold (at 65 °C) under the presence 0.025% of SDS, respectively [22]. It has been assumed that increased protein flexibility by anionic detergent stimulates the phosphotriesterase activities in PLL enzymes. In order to investigate this SDS-inducible activating effect on 4Mut, phosphotriesterase activity assay was performed in the presence of different concentrations of SDS using 500 μM paraoxon as substrate. As shown in Figure 4a, the paraoxonase activity of 4Mut has reached the highest value under the presence of 0.025% SDS. The residual activity was enhanced 3-fold compared with the condition without SDS. On the other hand, the activity decreased considerably with the SDS concentrations higher than 0.025%. In particular, in the presence of 0.1% SDS, the phosphotriesterase activity was reduced more than 60% compared with the condition without SDS. Accordingly we performed the kinetic characterization of 4Mut under the presence of 0.025% SDS at both 25 and 70 °C (Table 2). Detailed kinetic analyses revealed that catalytic efficiency (*k*_cat_/K_M_) at 70 °C reached 4.3 × 10^5^ M^−1^ s^−1^, exhibiting 2.6-fold enhancement under the presence of 0.025% SDS. Both lowered K_M_ and increased *k*_cat_ by SDS were found to be responsible for the improved *k*_cat_/K_M_ value. At 25 °C, while *k*_cat_ value was improved slightly, K_M_ value was reduced approximately 2-fold under the presence of 0.025% SDS, resulting in 2-fold improvement in *k*_cat_/K_M_ (1.0 × 10^5^ M^−1^ s^−1^).

We further investigated the stability of 4Mut by incubation in the presence of 0.025% SDS at room temperature for certain period of times (Figure 4b). 4Mut retained more than 80% and 40% of its paraoxonase activity after incubation for 2 and 4 weeks at room temperature, respectively. These results indicate that 4Mut possesses a substantial resistance on SDS.

#### 2.3.3. Lactonase and Carboxylesterase Activities of 4Mut

In contrast to the improved hydrolytic activities on phosphotriesters, 4Mut shows no catalytic activities on carboxylesters including pNP-butanoate and β-naphthyl acetate. High catalytic activity on synthetic lactone TBBL (5-thiobutilγbutyrolactone) was detected (63.0 U/mg), however, there was no activity on hydrolysis of C8-HSL and C10-HSL (N-octanoyl and N-decanoyl homoserine lactones). Overall, 4Mut possesses similar substrate profile with 3Mut [22].

#### 2.3.4. Detoxification of Nerve Agents

We previously demonstrated the ability of *Sso*Pox, W263F and *Sac*Pox to hydrolyze a deadly nerve agent, cyclosarin [19]. In addition, we have recently reported the detoxification of other chemical warfare nerve agents including tabun, sarin, soman and cyclosarin using 3Mut [33]. It is of great interest to test the ability of 4Mut to hydrolyze these nerve agents. Table 3 shows the catalytic efficiency of 4Mut against the hydrolysis of tabun, sarin, soman, cyclosarin and VX at 37 °C. Even though 4Mut was obtained through the screening against paraoxon, the enzyme has exhibited 10-fold enhancement in the hydrolysis of tabun and soman with respect to 3Mut. On the other hand, single mutation introduced in 3Mut resulted in complete loss of activity against cyclosarin.

### 2.4. 3D Structure of 4Mut

**Figure 5:**
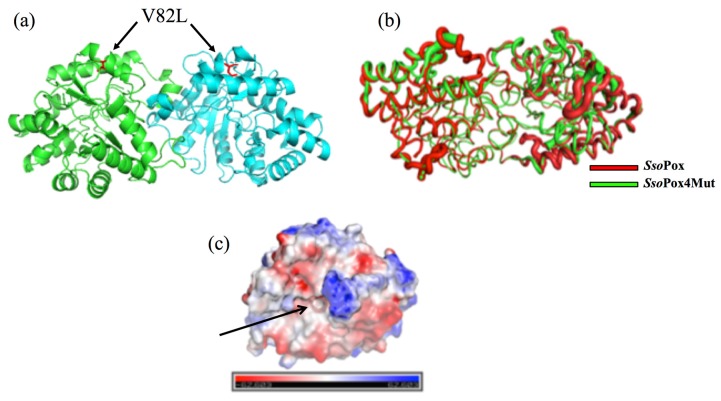
Structure of 4Mut. Panel (**a**) shows a cartoon of the 4Mut dimer in which the two monomers are colored differently (green and blue). Mutated V82L residues in both monomers are highlighted in red. In panel (**b**) is shown a superposition of 4Mut (green) and wt (red) tubes colored by B-factors highlighting the presence of more flexible regions in the mutants (ticker tube). Panel (**c**) is a representation by electrostatic potential surface coloring showing the “hole” of the active site indicated by an arrow.

**Figure 6:**
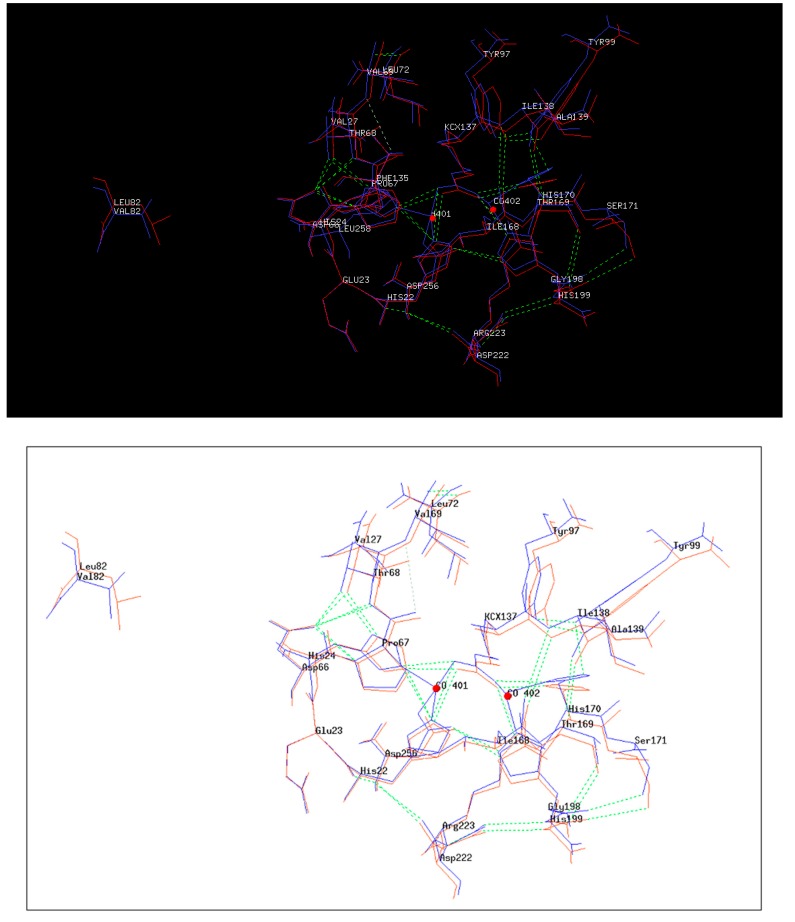
Details of the actives sites. In Figure 6 details of residues (stick representation) of the active sites of 3Mut (blue) and 4Mut (red) are given. Residues within 8 A from CX of K137 are displayed. The Co^2+^ ions in 4Mut are shown as red spheres.

3D structure of 4Mut was obtained by the hanging drop method as described in Methods. At first we got crystals from 10% PEG 3000 0.05 M HEPES pH 7.5. These crystals did not diffract at high resolution. Thus we used seeds of these crystals and got crystals using the following conditions: 10% PEG 3000, 0.05 M HEPES pH 7.5, 0.1 M NaCl. These crystals were obtained by utilizing the sitting drop vapor diffusion method, at 19°C and diffracted at 2.8 Å (Figure S7). Details of refinement statics of 4Mut crystal structure are summarized in Table 4. Figure 5a shows the dimeric structure of 4Mut protein. In Figure 5b, 4Mut (green) and wt (red, PDB code: 2VC7) structures were represented by B-factors and superimposed. It should be noted that 4Mut possesses more flexible regions as shown by higher B-factor values, suggesting the introduction of conformational changes due to the four mutations. The hole of the active site was visible by 4Mut monomer surface representation colored by electrostatic potential (Figure 5c). Furthermore, Inductively Coupled Plasma mass spectrometry (ICP-MS) analysis revealed the presence of two cobalt atoms in the protein in agreement with the metal added in the *E. coli* culture for the enzyme preparation. This is in contrast with the previous result in which *Sso*Pox was shown to possess heterobinuclear metal center constituted by an iron at α-site and a cobalt cation at β-site after purification in the presence of Co^2+^ ion in the buffer [34]. However, in a previous work we demonstrated the possibility to change the metal content in PLL members with sometimes significant changes in enzyme properties [21]. The details of active sites of 3Mut (Figure 6; blue) and 4Mut (Figure 6; red) were shown with metal cofactors (Co^2+^; red spheres) detected. The RMS difference between 3Mut and 4Mut was 0.59 Å on 604 superimposed C_alpha_ atoms. In 4Mut, many small changes with respect 4Mut were visible. In particular an hydrogen bond between Tyr99 and His 170 coordinating one of Co^2+^ ions was missing. Many residues were shifted with respect the 3Mut. That suggests a different conformation imparted by mutations.

### 2.5. Structures Comparison

**Figure 7:**
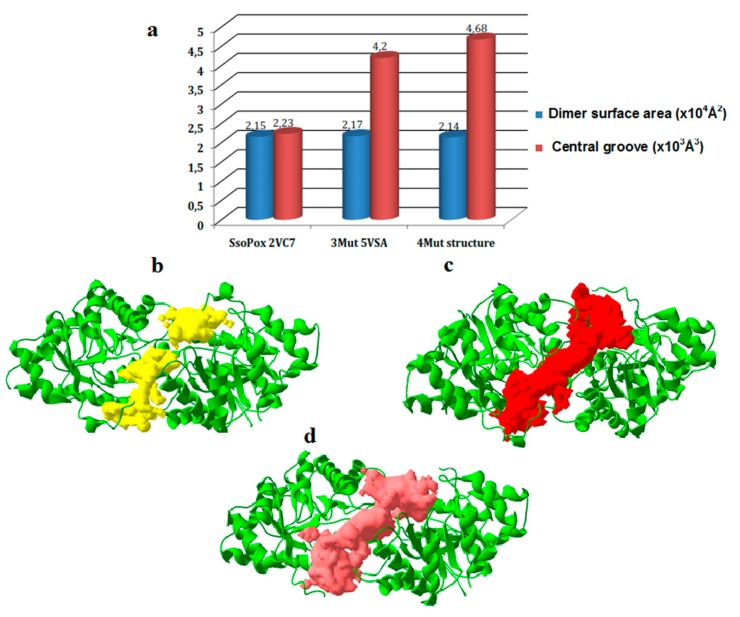
Analyses among *Sso*Pox, *Sso*Pox 3Mut and 4Mut. (**a**) Dimer surface area and central groove volume of (**b**) *Sso*Pox 3D structure (PDB: 2VC7), (**c**) 3Mut structure (PDB: 5VSA and (**d**) 4Mut 3D structure.

Surprisingly, the single mutation introduced in 4Mut confirmed by the structure is not located in the vicinity of active site (Figure 5a). In order to interpret the positive effect of this mutation on catalytic activity, we performed a comparative analysis on both the interface of two monomers and dimer surface area among *Sso*Pox, 3Mut and 4Mut (Figure 7). The dimer of previously solved 3D structure of *Sso*Pox (PDB code: 2VC7) exhibited two large grooves at the interface of two monomers. Importantly, amino acid residues lined along these cavities include Val 27, Leu 72, Tyr 97, Ile 98, and Trp 263, which were all previously reported in the mutagenesis study to increase the phosphotriesterase activity of the enzyme because of their involvement in the catalysis based on the comparison with PTE [19,22,34]. The 3D structure of 3Mut was recently reported [30]. In the structures of 3Mut and 4Mut, the central cavities observed in the *Sso*Pox structure appeared as a large groove alongside the interface of two monomers (Figure 7c,d). We performed the comparison of this central groove volume and dimer surface area between *Sso*Pox, 3Mut and 4Mut (Figure 7a). The central groove and dimer surface in *Sso*Pox structure were calculated as 2.23 × 10^3^ Å^3^ and 2.15 × 10^4^ Å^2^, respectively. Interestingly, three amino acids mutations resulted in the substantial increase in the central groove volume, reaching at 4.20 × 10^3^ Å^3^. The fourth mutation has further induced the enlargement of the central groove, showing 4.68 × 10^3^ Å^3^ (4Mut structure). Since central groove is located at the interface of two monomers in an enzyme molecule, the larger space between two monomers can be interpreted as higher flexibility of the dimer. From this view, we could conclude that *Sso*Pox has the lowest dimer flexibility among the three enzymes, with more compact, rigid conformation. Both mutations introduced in 3Mut and 4Mut showed positive effects on the central groove volume, therefore on dimer flexibility.

### 2.6. Circular Dichroism Analyses on 4Mut and 3Mut

It is of great interest to investigate how a single mutation located far from the active site affected the paraoxonase activity. Our hypothesis is that, single mutation at position 82 has slightly altered the overall architecture of the enzyme, which resulted in the improved catalytic activity. We further investigated the structural organization of 3Mut and 4Mut by far- and near-UV circular dichroism analyses.

#### 2.6.1. Far-UV CD Spectra for the Analysis of Secondary Structures

**Figure 8:**
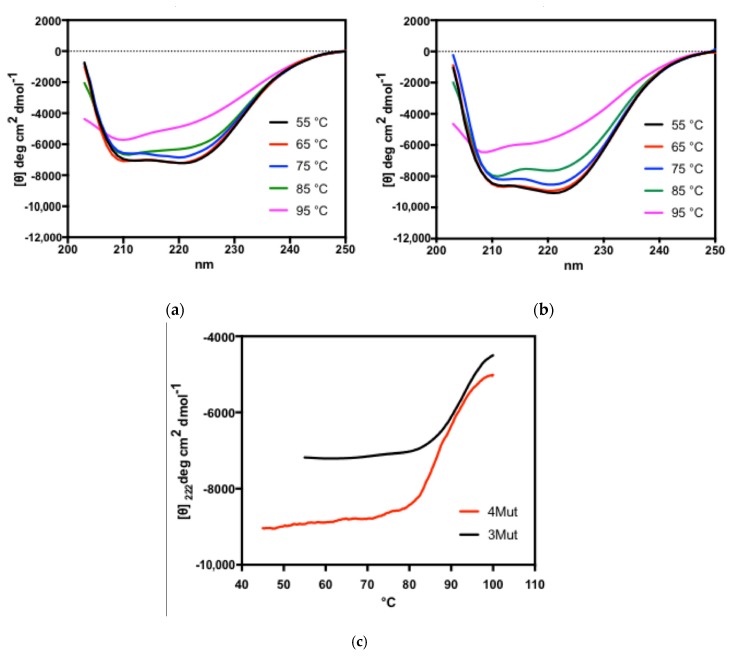
Far-UV CD spectra of (**a**) 3Mut and (**b**) 4Mut. The CD spectra were obtained at 55, 65, 75, 85 and 95 °C. (**c**) Thermal denaturation curves of 3Mut (black line) and 4Mut (red line). Thermal denaturation curves were measured by recording the molar ellipticity at 222 nm, and melting temperatures (Tm) for 4Mut and 3Mut were determined as 86.7 °C and 92.3 °C, respectively.

The far-UV CD spectra of 3Mut and 4Mut are shown in Figure 8a,b. Both native enzymes (at 25 °C) exhibit two negative peaks at 208 and 222 nm as reported in the spectrum of *Sso*Pox, suggesting that two mutant enzymes possess a large alpha-helical content as wild-type enzyme does, and that mutations did not collapse the secondary structure of the enzyme [35]. At 75 °C, both enzymes showed slightly altered far-UV CD spectra compared with the native enzymes, indicating that the secondary structure of the enzymes have been disrupted at this temperature. At the highest temperature tested (95 °C), we observed a large loss of secondary structures in two enzymes, as both mutants showed completely different far-UV CD spectra from native ones.

For further investigation on the conformational stability of two enzymes at the secondary structure level, we acquired thermal denaturation curves by recording the molar ellipticity at 222 nm (Figure 8c)**.** Thermal denaturation curves revealed that denaturation temperatures for 4Mut and 3Mut were 86.7 and 92.3 °C, respectively. 3Mut exhibited higher melting temperature than 4Mut by 5.6 °C, however, these values are much lower than that of *Sso*Pox, which was previously determined as 106 °C [35].

#### 2.6.2. Near-UV CD Spectra for the Analysis of Tertiary Structures

**Figure 9:**
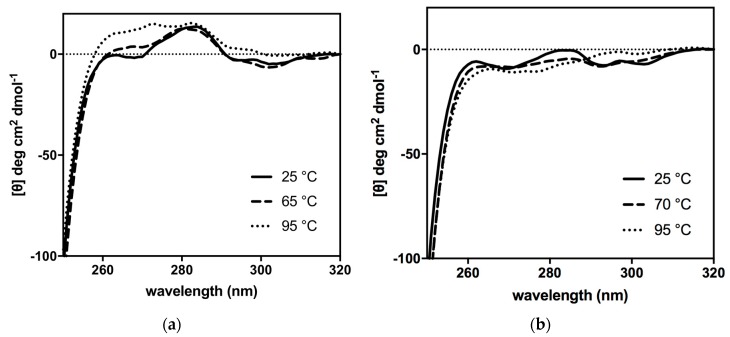
Near-UV CD spectra of (**a**) 3Mut and (**b**) 4Mut. The CD spectra were obtained at 25, 65 and 95 °C for 3Mut, and 25, 70 and 95 °C for 4Mut.

CD spectra at near-UV region were also acquired for both 3Mut and 4Mut at different temperature conditions (Figure 9). It is notable that different elements associated to the tertiary organization of each enzyme have been detected. In the spectra for 4Mut, a positive band centred at about 284 nm exhibited substantial changes in response to rise in temperature (Figure 9b). In contrast, the spectra at the same region in 3Mut remained almost unaffected by the temperature increase (Figure 9a). Instead, a certain change in the region at around 270 nm was detected in 3Mut spectra, with the appearance of a positive signal particularly at 95 °C. 4Mut showed no significant CD signal around 270 nm at all temperatures examined. Comparison on the near-UV CD spectra at 25 °C between two mutant enzymes implied that two mutants are endowed with different conformation of the tertiary structures at native forms. Probably, single mutation in 4Mut has introduced a slightly altered tertiary structure, therefore resulted in a different organization of quaternary structure. Furthermore, near-UV CD spectra reflect the different behaviours of two mutants towards temperature increase.

## 3. Discussion

We utilized three homologous PLL genes encoding *Sso*Pox 3Mut (C258L/I261F/W263A), GKL and Dr0930 to generate new mutant PLL variants through DNA StEP method. Two bacterial PLL genes, *gkl* and *dr0930*, show quite low sequence identities with respect to C258L/I261F/W263A; 35.8% for gkl and 29.9% for dr0930, respectively. In principle, DNA StEP method requires homologous genes with more than 80% sequence identities for recombination [31]. Therefore, it was challenging to generate new variants utilizing homologous genes with such low sequence identities. Indeed, sequence analysis on newly obtained 4Mut variant revealed an extremely low rate of template switching during gene recombination by DNA StEP reaction, with only single mutation introduced in comparison with 3Mut. Substitution of valine residue into leucine at position 82 was accomplished by only a nucleotide alteration. Since no template genes possess leucine at the corresponding position, it is unclear how this mutation in 4Mut was introduced during DNA StEP recombination. One explanation could be the generation of loop structure in either template genes or annealed DNA fragments during gene recombination reaction, due to base-base mismatching.

In the course of molecular evolution from *Sso*Pox to 3Mut, we observed a weak trade-off in the specificity between main lactonase activity and promiscuous paraoxonase activity. Three mutations brought about a 9-fold decrease in lactonase activity on TBBL, while paraoxonase activity increased 292-fold. This led to the paraoxonase activity in 3Mut exhibiting approximately 3-fold higher than the lactonase activity on TBBL, in contrast to the wt, which showed 760-fold lower paraoxonase activity than its main lactonase activity [22]. In the case of the engineering from 3Mut to 4Mut, alteration of co-existing two catalytic activities proceeded in the same direction; both lactonase and paraoxonase activities were improved 2-fold, almost retaining the same substrate profile observed in 3Mut [30]. It is likely that upon selective pressure on the new phosphotriesterase activity, concurrent alteration in native lactonase activity appears rather random and independent from improved new activity, which is consistent with the proposal recently made by Kaltenbach and colleagues [36].

As shown in Table 1, 4Mut exhibited hydrolytic activity against m-parathion, but not on (ethyl) parathion. In contrast, hydrolytic activity of 4Mut against (ethyl) paraoxon was approximately 4-fold higher than that against m-paraoxon. Crystal structures of 3Mut (PDB:5VSA) and other *Sso*Pox variants suggested that conformational rearrangement in active site loop 8, which is involved in substrate binding, is strongly correlated with substrate specificity spectrum [30]. Destabilization of loop 8 in 3Mut was subtle compared with other variants showing broader substrate specificity. Comparable substrate profile between 3Mut and 4Mut can be due to the similar loop 8 conformation in two variants. In addition, since 4Mut has been screened using paraoxon as substrate, we selected a new variant showing high hydrolytic activity particularly against paraoxon.

On the other hand, we observed that single mutation introduced in 4Mut has resulted in altered enzymatic and biochemical properties compared with 3Mut. First of all, kinetic comparisons of paraoxonase activities between 4Mut and 3Mut at two temperatures (25 °C and either 65 or 70 °C) pointed out some interesting differences. In fact, even though *k*_cat_/K_M_ values for the paraoxonase activity of both 4Mut and 3Mut increased at higher temperature with respect to those at 25 °C, different factors are involved in their enhancement. In case of 4Mut, the substrate affinity (K_M_) did not show significant changes between 25 and 70 °C. The higher *k*_cat_/K_M_ value at 70 °C was therefore mainly due to the increased *k*_cat_. In contrast, K_M_ in 3Mut at 25 °C showed a significantly reduced value compared with that obtained at 65 °C. However, specific activity (V_max_) of 3Mut at 25 °C was considerably low (8.74 U/mg) compared with the value at 65 °C (123.6 U/mg), leading to the low *k*_cat_ for 3Mut at 25 °C (6.91 s^−1^). It seems that positive effect of K_M_ value on catalytic efficiency in 3Mut was masked by low *k*_cat_ at 25 °C. In addition, it should be noted that 4Mut showed approximately 3-fold increased *k*_cat_/K_M_ values in comparison with 3Mut at their optimal temperature (70 °C or 65 °C) and 25 °C. At high temperatures, both improved substrate affinity and *k*_cat_ have contributed to the enhanced catalytic efficiency in 4Mut. By contrast, at 25 °C, even though 3Mut exhibited better K_M_ compared with 4Mut, *k*_cat_ was almost 5-fold lower than the value in 4Mut. Therefore, in comparison with 3Mut, high *k*_cat_ in 4Mut seems the primary factor in enhanced *k*_cat_/K_M_ at 25 °C. Furthermore, kinetic data obtained in the presence of SDS revealed that 4Mut responded in a different way to the 0.025% SDS at 70 °C with respect to 3Mut. Even though 0.025% SDS enhanced the catalytic efficiency of 3Mut by 2-fold at 65 °C, improved *k*_cat_/K_M_ was mainly due to a significant improvement of the substrate affinity that compensated a large reduction in the *k*_cat_ value.

From the biochemical point of view, 4Mut exhibited a characteristic pattern of thermophilicity. 4Mut did not show a clear optimal temperature, but rather maintained high activity in the temperature range between 55 °C and 70 °C. On the other hand, 3Mut showed a clear optimal temperature at 65 °C, with a substantial activation effect by increased temperature up to 65 °C [22]. This heat-induced improvement in the hydrolytic activity in 3Mut seems accompanied by a decrease in substrate affinity, whereas 4Mut did not show such a phenomenon. In combination with the results of thermophilicity curves and near-UV CD spectra in both 3Mut and 4Mut, we hypothesize that these two variants possess distinct tertiary structures thus resulting in different quaternary organizations of the enzymes at native level. The near-UV CD signal at around 270 nm found in 3Mut might be associated with the active form of this mutant. Indeed, this signal was absent at 25 °C in which the enzyme was in a less active form. By increasing temperature up to 65 °C, 3Mut underwent a change in its tertiary structure associated to the above-mentioned positive signal, resulting in the increased activity for 3Mut enzyme. In case of 4Mut, it seems that thermal treatment provokes modifications of the tertiary structure in a different way. Probably, native 4Mut is already in an active form, as observed in its thermophilicity curve showing the maintained high catalytic activity in a wide temperature range (Figure 3b). Increased temperature up to 70 °C did promote distinct tertiary structural rearrangement from the structural modification observed in 3Mut, which led to a slight improvement in enzymatic activity. The change of tertiary structure in 3Mut involves probably phenylalanine residues accounting for the CD signals at about 270 nm. By comparing the accessibility of Phe in 4mut and 3Mut we observed that only Phe104 in 3Mut was more accessible.

In case of 4Mut, the rearrangement of tertiary structure can be due more probably to tryptophan and tyrosine moieties, which influence the CD signals at about 284 nm.

Two residues have a different accessibility in this case, namely Trp36 and Tyr40. From far- and near-UV CD analyses, we could conclude that molecular evolution from 3Mut to 4Mut involving an improvement in paraoxonase activity is accomplished by structural rearrangement at tertiary structure level, which thus affects the quaternary organization, without significantly affecting the secondary organization of the enzyme.

From the thermal stability analyses, it was revealed that 4Mut retains half of its paraoxonase activity for 16.7 h at 65 °C, and 8.4 h at 70 °C. At 75 and 80 °C, the t_1/2_ values were determined as 3.3 h and 2.1 h, respectively. Previously characterized single mutant of *Sso*Pox, W263F, showed much lower thermal stability, with t_1/2_ values as 3.3h at 70 °C and 1.5h at 80 °C [19]. On the other hand, since 3Mut showed no activity changes at 65 °C and 75 °C, it is likely that 4Mut is less resistant to high temperature compared with 3Mut. Slightly decreased thermal resistance in 4Mut was also evident in thermal denaturation curves obtained by recording the molar ellipticity at 222 nm in far-UV CD measurement. The melting temperature of 4Mut was determined as 86.7 °C while 3Mut exhibited 92.3 °C, which is 5.6 °C higher than 4Mut (Figure 8c). It is important to note that, even though these values are lower than the previously determined melting temperature of *Sso*Pox (106 °C), they are substantially higher than that for PTE from *Brevundimonas diminuta* MG (75 °C) [35].

The comparison of the volume of central groove in the models revealed that *Sso*Pox exhibited the lowest value among *Sso*Pox, 3Mut and 4Mut (Figure 7a). This result indicates that the dimer of *Sso*Pox is the most rigid and compact, therefore with the lowest flexibility among three enzymes. The enlargement of central groove was observed according to the mutations introduced in both 3Mut and 4Mut, thus possibly 4Mut possesses the highest dimer flexibility. We believe that the tighter dimer conformation in *Sso*Pox has provided *Sso*Pox with the highest thermal resistance among three enzymes. Subsequently, three mutations in 3Mut resulted in substantial decrease in thermal resistance, which was reflected by melting temperature and thermal stability experiments as a negative impact of increased dimer flexibility. Further mutation in 4Mut led to the increased dimer flexibility, in turn, reduced thermal resistance. This trend is in agreement with our previous study on the determinants of high thermal stability in *Sso*Pox, through structural comparison with its mesophilic counterparts, PTE from *Brevundimonas diminuta* MG and OpdA from *Agrobacterium radiobacter* [35]. It was revealed that *Sso*Pox possesses a tighter quaternary structure because of the optimization of the interactions at the interface between two monomers. Compact, and rigid dimer formation of *Sso*Pox was strongly suggested as one of the major determinants for the exceptional thermal stability observed in *Sso*Pox. We have therefore demonstrated again the strong correlation between dimer conformation and thermal stability.

In addition, we further observed that increased flexibility in dimer conformation has led to increased catalytic activity on paraoxon. Even though *Sso*Pox has the highest stability with its melting temperature at 106 °C, the hydrolytic activity of *Sso*Pox is lowest among all enzymes described above [35]. Flexibility is one of the key factors for catalytic activity whereas rigidity is necessary to retain the stability. This could be applied for the dimer conformation of PLL enzymes as well as PTE. One of the explanations for correlation between catalytic activity and dimer conformation could be that, central cavity located at the interface of two monomers of *Sso*Pox was found to connect two active sites in the enzyme molecule. Interestingly, amino acid residues located along this cavity include Val 27, Leu 72, Phe 97, Ile 98, and Trp 263. All these positions were previously reported in the mutagenesis study to increase the phosphotriesterase activity of the enzyme because of their involvement in the catalysis based on the comparison with PTE [19,22,34]. Therefore, it is a strong indication that alteration in dimer conformation observed in 4Mut could have a substantial impact on the flexibility of active site through enlargement in central cavity volume. We propose that reorganization of dimer conformation can be the major determinant for the improvement in hydrolytic activity in 4Mut through increased central cavity volume and dimer flexibility. This hypothesis is further supported by the results of SDS-inducible activation effect on the catalytic activity of PLL enzymes; anionic detergents such as SDS have demonstrated to enhance paraoxonase activity of PLL enzymes. SDS may increase enzyme flexibility through breaking hydrogen bonds in the enzymes, resulting in altered dimeric conformation in PLL enzymes [37,38,39].

## 4. Materials and Methods

### 4.1. Chemicals

Organophosphate pesticides used in this study were purchased from Sigma Chemical Co. (St. Louis, MO, USA). Molecular mass markers for SDS-PAGE, restriction enzymes were obtained from Bio Rad (Hercules, CA, USA) and New England BioLabs (Beverly, MA), respectively. All other chemicals were purchased from Sigma Chemical Co. (St. Louis, MO, USA). Chemical nerve agents including tabun (GA), sarin (GB), soman (GD), cyclosarin (GF) and VX were provided by the Bundeswehr Institute of Pharmacology and Toxicology (Munich, Germany).

### 4.2. DNA Manipulation

Standard molecular cloning techniques were employed. *E. coli* strains TOP10 or BL21(DE3) were grown in Luria-Bertani (LB) medium containing either ampicillin (100 mg/mL) or kanamycin (50 mg/mL) at 37 °C. Purification of plasmid DNA was performed using QIAprep Miniprep or Midiprep kits (Qiagen, Hilden, Germany). The concentration of DNA was determined by NanoDrop 1000 (Thermo Fisher Scientific, USA).

### 4.3. Preparation of PLL Genes as Templates for DNA StEP

Cloning of *sislac* gene was performed as described below. A 945-bp of *sislac* gene fragment was amplified by PCR reaction using genomic DNA of *Salfolobus islandicus* M 16.4 (kindly provided by Dr. Rachel Whitaker, University of Illinois) as template. 300 ng of genomic DNA of *S. islandicus* M 16.4 was mixed with the PCR reaction solution containing 2 μM 5′ sislac oligonucleotide (5′-CTATTATCATATGAGAATACCATTAGTTGGG-3′), 2 μM 3′ sislac oligonucleotide (5′-ATATAAGCTTTTAGCTGAAGAATTTTTTCGG-3′), 200 μM dNTP, 2.0 mM MgSO4, 1U Vent DNA polymerase in the buffer conditions suggested by the manufacturer. The primer 5′ sislac was designed to introduce an NdeI restriction site upstream of the sislac gene, whereas primer 3′ sislac was designed to introduce a HindIII restriction site downstream from the stop codon of *sislac* gene. The amplification of *sislac* gene was performed by the PCR programme as follows: 2 min at 95 °C, followed by 30 cycles of 95 °C for 2 min, 58 °C for 1 min and 72 °C for 1 min. The amplified *sislac* gene was digested by NdeI and HindIII restriction enzymes, and purified by QIAquick PCR purification kit (Qiagen, Germany). Obtained DNA fragments were ligated into the vector pT7-7 (a derivative of pBR322). Vector pT7-7 was treated with Alkaline Phosphatase, Calf Intestinal (CIP), prior to ligation reactions. Chemical competent *E. coli* TOP10 (Stratagene) was transformed with ligated plasmid DNA and subsequently plated on LB agar plate containing 100 μg/mL ampicillin. Obtained plasmid DNA pT7-7-*sislac* was sequenced to verify the insertion of *sislac* gene.

The gene encoding *vmolac* was optimized for the expression in *E. coli* and cloned into vector pEX-A2 by Eurofins Genomics, Germany. NdeI and HindIII restriction sites were introduced upstream and downstream of *vmolac* gene, respectively. Chemical transformation of pEX-A2-vmolac into *E. coli* (TOP10) was carried out and plasmid DNA pEX-A2-vmolac was obtained through Midiprep kits (Qiagen, Hilden, Germany).

The plasmid DNA pT7-7-sacpox was generated as described in Porzio et al. 2007 [20]. Plasmid DNA pT7-7-*C258L/I261F/W263A* and pT7-7-*dr0930* constructs were obtained as described in Del Giudice et al. 2016 [22] and Mandrich et al. 2013 [40], respectively. pET 28a (+)-*gkap(gkl)* construct was kindly donated by Yan Feng from Shanghai Jiao Tong University, China.

pT7-7-*C258L/I261F/W263A* construct was digested by NdeI and EcoRI restriction enzymes, while NdeI and BamHI were utilized to digest pT7-7-*dr0930.* For the digestion of pT7-7-*dr0930,* BamHI and HindIII were used. pT7-7-*sislac* and pEX-A2-*vmolac* were digested with NdeI and HindIII. pT7-7-*sacPox* was digested with NdeI and PstI. Digestion for vector-gene construct was performed at 37 °C for 1.5 h. Digested gene fragments (approximately 1000 bp) were gel-purified by QIAquick Gel Purification kit (Qiagen, Germany). Purified gene fragments were eluted with 40 µl sterile milliQ water and stored at −20 °C.

### 4.4. Generation of Mutant Library by DNA StEP

1.0 ng of digested and purified PLL genes were mixed in the reaction solution containing 200 µM dNTP mix, 2.0 mM MgSO_4_, 1U Vent DNA polymerase in appropriate buffer condition, 1 µM 5′ oligonucleotide (5′-CTATTATCATATGAGAATACCATTAG TTGGG-3′) and 1 µM 3′ oligonucleotide (5′-ATATAAGCTTTTAGCTGAAGAATTT TTTCGG-3′). The 5′ primer was designed to introduce an NdeI restriction site upstream of the *C258L/I261F/W263A* gene, whereas 3′ primer was designed to introduce a HindIII restriction site downstream from the stop codon of *C258L/I261F/W263A* gene. Total volume was adjusted at 50 µl by adding sterile milliQ water. Thermal cycle of DNA StEP programme included denaturation at 94 °C for 30 s, followed by extremely abbreviated annealing/polymerase-catalyzed extension at 55 °C 2 s. In order to obtain full-length mutant gene, the thermal cycle was repeated 50 times.

### 4.5. Expression of Mutant Library

Recombinant product obtained through DNA StEP reaction was digested by DpnI restriction enzyme in order to eliminate template genes, and purified by QIAquick PCR purification kit (Qiagen, Germany). 100 ng of purified DNA fragments were amplified by PCR in a reaction solution containing 2 µM 5′ oligonucleotide (5′-CTATTATCATAT GAGAATACCATTAGTTGGG-3′), 2 µM 3′ oligonucleotide (5′-ATATAA GCTTTTAGCTGAAGAATTTTTTCGG-3′), 200 µM dNTP, 2.0 mM MgSO_4_, 1U Vent DNA polymerase in the buffer conditions suggested by the manufacturer. The amplification was performed by the PCR programme as follows: 2 min at 95 °C, followed by 30 cycles of 95 °C for 2 min, 58 °C for 1 min and 72 °C for 1 min.

Amplified mutant library were then digested with NdeI and HindIII restriction enzymes. After purification using QIAquick PCR purification kit, mutant library were ligated into the vector pT7-7 which was treated with Alkaline Phosphatase, Calf Intestinal (CIP) prior to ligation reactions. 10 ng of ligations were used to transform 40 µL of electrocompetent *E. coli* BL21 (DE3) by electroporation using the MicroPulser electroporator (BIO-RAD) with the voltage of 2.5 kV. Transformed *E.coli* BL21 (DE3) was subsequently plated on LB agar plate containing 100 µg/mL ampicillin.

### 4.6. Screening for Paraoxonase Activity of Variants

Each colony on LB agar plates was picked up to inoculate 200 µL of LB medium containing 100 µg/mL ampicillin in the 96-well cell culture sterile plates. The plates were incubated for overnight at 37 °C and mutant PLL enzyme expression was induced for 3 h at 37 °C after addition of IPTG with the final concentration of 1 mM. For the cell lysis, 80 µL of cell culture was mixed with 20 µL of lysis buffer containing 0.1 M Tris-HCl (pH8.0) and 0.25% SDS in a new 96-well cell culture sterile plate, and incubated at 4 °C for 1 h. Identical two 96-well plates were prepared by transferring 50 µL of lysate solution into a new 96-well plate for the duplication of paraoxonase activity assay. 150 µL of activity assay solution was added to lysis solution with the final concentration of 20 mM Tris-HCl (pH8.0 at 70 °C) and 1 mM paraoxon as substrate. The absorbance at 405 nm was measured after incubation for 30 min at 70 °C, in order to detect the production of *p*-nitrophenol, one of the hydrolysis products of paraoxon.

### 4.7. Protein Expression and Purification in Large Scale

3 mL of LB medium with 100 µg/mL ampicillin was inoculated with single colony, and incubated at 37 °C for 10 h. The 3 mL pre-culture was used to inoculate 1.5 L of LB medium containing 100 µg/mL ampicillin and cells were grown at 37 °C for overnight. IPTG (0.5 mM) and CoCl_2,_ (0.5 mM) were added to the growth medium and incubated at 37 °C for 4 h. Cells were harvested by centrifugation at 6000 rpm for 15 min at 4 °C, and pellets (4.5 g) were stored at −20°C. Wet frozen cells were thawed and resuspended in 25 mL of buffer A (20 mM HEPES-NaOH pH 8.5, 0.5 mM CoCl_2_) and lysed by sonication (Branson). The programme for sonication was set as follow; 10 s ON, 10 s OFF, total time 3 min, 30% intensity. Cell debris was removed by centrifugation at 80,000 *g* for 30 min at 4 °C. Crude extract (25 mL) was incubated at 60 °C and 70 °C for 15 min under gentle stirring, in order to thermoprecipitate proteins derived from *E. coli* and separated by centrifugation at 80,000 *g* for 30 min at 4 °C between each thermoprecipitation step. Subsequently anion exchange chromatography was performed for further purification. Enzyme solution obtained through 70 °C incubation was loaded onto Q-Sepharose Fast Flow FPLC column (Pharmacia) equilibrated with buffer A, connected to an AKTA pure system (GE Healthcare). Following the wash with buffer A, a linear gradient of NaCl (0–0.5 M) was applied for the elution of the proteins with the flow rate 1 mL/min. The fractions were analyzed by both SDS-PAGE and phosphotriesterase activity assay using 1 mM paraoxon as substrate as described below. Fractions with activity on paraoxon were concentrated by ultrafiltration. Size-exclusion chromatography was performed using Superdex 75 column 16 × 60 cm^2^ (GE Healthcare) equilibrated with buffer containing 20 mM HEPES-NaOH (pH 8.5), 0.5 mM CoCl_2_ and 200 mM NaCl. The flow rate was set as 0.5 mL/min. The fractions were analyzed by SDS-PAGE and paraoxonase activity assay as described below, and stored at 4 °C.

### 4.8. Phosphotriesterase Activity Assay

Standard paraoxonase activity assay was performed essentially as described in Merone et al. 2005 [18]. In brief, enzyme was added in an assay solution containing 20 mM HEPES-NaOH (pH 8.0) and paraoxon as substrate. Hydrolysis of paraoxon was monitored by measuring the absorbance at 405 nm to detect the production of *p*-nitrophenol in 1 cm path-length cells using Cary 100 spectrophotometer (Varian, Australia). The molar absorption coefficients used for *p*-nitrophenol were reported previously [18]. Activities on other phosphotriesters were measured as described in Del Giudice et al. 2016 [22] using 1 mM of substrates. Kinetic parameters on paraoxon or methyl-paraoxon hydrolysis were determined essentially as described previously [22]. Kinetic parameters on paraoxonase activity were measured at 70 °C and 25 °C with and without 0.025% SDS, with the concentrations of paraoxon ranging from 10 µM to 2.0 mM. The pH was adjusted at 8.0 by 20 mM HEPES in the assay solution. Kinetic measurements for methyl-paraoxon hydrolysis were performed using solution containing 20 mM HEPES-NaOH pH8.0 at 70 °C, with varied concentrations of methyl-paraoxon from 10 µM to 1 mM.

### 4.9. Determination of Metal Ions for the Enhancement of Paraoxonase Activity

Effect of metal ions on paraoxonase activity of 4Mut was analyzed essentially described in Porzio et al. 2013 [21]. In detail, *E.coli* BL21(DE3) carrying plasmid pT7-7-4Mut were grown in 100 mL LB medium containing 100 µg/mL ampicillin at 37 °C for overnight. 10 mL aliquots of cell culture were prepared in new sterile tubes. IPTG with the final concentration of 0.5 mM and either ZnCl_2_, CoCl_2_, MnCl_2_, CdCl_2_ or NiSO_4_ (final concentration: 0.5 mM) were added prior to further incubation at 37 °C for 4 h. Cells were harvested by centrifugation at 5000 rpm for 15 min at 4 °C, and pellets were stored at −20 °C. Each wet frozen cell was thawed, resuspended in 500 µl of 20 mM HEPES-NaOH (pH 8.5) and lysed by sonication (Branson). The programme was set as follow; 10 s ON, 10 s OFF, total time 1 min, 20% intensity. Cell debris was removed by centrifugation at 13,000 rpm for 10 min at 4 °C. Crude extract was incubated at 70 °C for 10 min and precipitated proteins derived from *E. coli* were removed by centrifugation at 13,000 rpm for 10 min at 4 °C. The paraosonase activity assay was performed as described above using each enzyme solution prepared in the presence of different metal ions.

### 4.10. Determination of Optimal pH, Thermophilicity and Thermal Stability

The dependencies of paraoxonase activity on pH, temperature and thermal stability were measured as described in Merone et al. 2005 [18]. The temperatures ranged from 65 to 80 °C in 20 mM HEPES pH 8.0 for the determination of thermal stability.

### 4.11. Lactonase Activity Assay

Lactonase activities on N-octanoyl homoserine lactone (C8-HSL), N-decanoyl homoserine lactones (C10-HSL) and 5-thiobutylγbutyrolactone (TBBL) were measured as described in Porzio et al. 2013 [21].

### 4.12. Carboxylesterase Activity Assay

Activity assay on carboxylesters including *p*-nitrophenyl (*p*NP) butanoate and β-naphtyl acetate was performed as described in Merone et al. 2005 [18].

### 4.13. Preparation of 4Mut Enzyme for Crystallization

*E. coli* BL21 (DE3) transformed with pT7-7-4Mut was grown in 7 L of LB medium containing 100 µg/mL ampicillin at 37 °C overnight. Protein expression was induced by 0.5 mM IPTG. Cell harvesting and purification were performed as described in the section “Protein expression and purification in large scale”. In addition, in order to obtain pure enzyme with high yield, additional purification process was performed after size-exclusion chromatography; the fractions containing 4Mut, with approximately more than 90% purity, were selected and pooled. Thermal precipitation was performed at 70 °C for 30 min, followed by ultracentrifugation at 80,000× *g* for 1 h at 4 °C. Supernatant was diluted 10-times with buffer A in order to lower the concentration of sodium chloride in the protein solution and applied on MonoQ column (5/50 GL, GE Healthcare) equilibrated with buffer A, connected to an AKTA pure system. After washing with buffer A, proteins were eluted by a linear gradient of NaCl (0–0.5 M) with the flow rate 1 mL/min. Purity of obtained fractions was analyzed by SDS-PAGE and fractions with more than 95% purity were pooled. Pure enzyme solution was substituted with buffer solution containing 20 mM HEPES-NaOH (pH 8.5), 0.5 mM CoCl_2_ and 200 mM NaCl and concentrated through ultrafiltration. Pure 4Mut with final concentration of 6.0 mg/mL was used for crystallization.

### 4.14. Crystallization, Data Collection, Refinement and Structure Determination

*Sso*Pox-4Mut plated like monoclinic crystals were obtained using the sitting drop vapor diffusion method with a Mosquito robot (TTP LabTech) at 19 °C utilizing the precipitant 20% propanol (*v/v*), 20% Polyethylene glycol (PEG) 3000, 100 mM HEPES pH 7.5 and 0.2 M NaCl. The 4Mut crystals formed in the orthorhombic space group *P2_1_2_1_2_1_*, with two dimers per asymmetric unit. Data collection was performed under cryo conditions (100 K), on ID30A-3 at the European Synchrotron Radiation Facility (ESRF, Grenoble, France). All diffraction images were indexed and integrated using the Mosflm program [41], and the integrated reflections were scaled using the SCALA program [42]. Structure factor amplitudes were calculated using TRUNCATE [43] from the CCP4 program suite (Table 4). The structure of *Sso*Pox-4Mut was solved by molecular replacement with the program PHASER [44], using the refined homologous (99% sequence identity) structure of the *Sso*Pox from the *Saccharolobus solfataricus* (PDB-ID: 4KES); [29]. All steps of atomic refinements were performed with the CCP4/REFMAC5 program [45]. The model was built into 2mF_obs_-DF_calc_, and mF_obs_-DF_calc_ maps using COOT [46]. Refinement movements were accepted only when they produced a decrease in the R_free_ value. All models were optimized using PDB_REDO [47], and were evaluated with MolProbity [48]. Details of the refinement statistics of the 4Mut structure are described in Table 4.

### 4.15. Metal Contents Analysis

Metal content analysis was performed by ICP-MS as reported in Porzio et al. 2013 [21].

### 4.16. Detoxification of Nerve Agents

5 µL of 1.0 mg/mL 4Mut in 20 mM HEPES pH 8.0 200 mM NaCl was mixed with 300 µL of nerve agent (VX: 4 μM; VR: 1 μM; GF: 1 μM; GB: 15 μM;) in 0.1 M Tris-HCl pH 7.4 at 37 °C. 50 µL aliquot was added in cuvette containing 3000 µL of 0.1 M phosphate buffer pH7.4, 100 µL of 10 mM DTNB and 50 µL of 28.4 mM acetylthiocholine at different times (5 to 60 min). The reaction was started by adding 10 µL of human acetylcholinesterase (AChE). The absorbance was followed for 5 min and the inhibition curves were analysed by non-linear regression analysis to get the first order inhibition rate constant k_1_ which was then plotted versus time to get the detoxification constant *k*_obs_. Data were corrected for spontaneous OP hydrolysis and finally *k*_cat_/K_M_ was calculated. Assays were performed in duplicate, and results were the means of two independent experiments.

### 4.17. Model Generation and Analyses

Structural comparison and cartoon representations of protein structures were performed using PyMOL system [49]. Root mean square deviations (rmsd) and analyses of surfaces and cavities were computed using Deep/View Swiss-Pdb-Viewer v. 4.1.0. [50].

### 4.18. Circular Dichroism

Far-UV and near-UV CD spectra were recorded with JASCO J-715 spectropolarimeter according to the procedure described previously in the literature [51,52] with minor modifications. The temperature was controlled with JASCO PTC-4235 equipped to the instrument. Cuvette of 1.0 cm path length was used for all the measurements. The protein concentrations were adjusted at about 0.015 mg/mL and 0.8 mg/mL for far-UV and near-UV CD spectra, respectively. Signals were recorded with 1.0 nm band width and 1.0 nm data pitch. Scans were triplicated for averaging. Baseline correction was performed through subtraction of a spectrum of buffer (10 mM Phosphate buffer pH 7.3).

## 5. Conclusions

Our results suggested a close relationship between dimerization, thermal stability and enzymatic activity for this class of enzymes. Also, a question on the mechanisms for the catalytic activity of PLL enzymes has emerged; if proper dimeric conformation is crucial for the catalytic activity of PLL enzymes, dimer or interface of monomers could provide a driving force for the activity. In other words, formation of dimer might be indispensable for this class of enzyme to retain their activity. This has highlighted the importance of mutagenesis approach that considers not only the active site of single monomer, but also effect on dimerization.

## Figures and Tables

**Table 1:** Specific activities of 4Mut on phosphotriesters, carboxylesters and lactones.

Class of Substrates	Name	λ (nm)	U/mg
Phosphotriester	paraoxon	405	206.4 ± 18.3
	m-paraoxon	405	50.9 ± 12.0
	parathion	405	N.A
	m-parathion	405	0.24 ± 0.01
	diazinon	228	N.A
	coumaphos	348	N.A
	dursban	276	N.A
	malathion	405	N.A
	phosmet	405	N.A
	dimethoate	405	N.A
	tolclophos	224	N.A
	cyanophos	246	N.A
Carboxylester	pNP-butanoate	405	N.A
	β-Naphthyl acetate	320	N.A
Lactone	N-octanoyl HSL	572	N.A
	N-decanoyl HSL	572	N.A
	TBBL	412	63.0 ± 3.4

**Table 2:** Kinetic parameters of *Sso*Pox and its variants.

Enzymes	Substrate	Conditions	V_max_ (U/mg)	*k*_cat_ (s^−1^)	K_M_ (mM)	*k*_cat_/K_M_ (s^−1^ M^−1^)
***Sso*Pox** ^[18]^	paraoxon	70 °C	0.42 ± 0.02	0.24 ± 0.01	0.060 ± 0.009	3.8 ± 0.75 × 10^3^
**W263F** ^[19]^	paraoxon	70 °C	12.8 ± 0.3	3.8 ± 0.14	0.153 ± 0.039	2.4 × 10^4^
**W263F** ^[19]^	paraoxon	25 °C	n.d.	n.d.	n.d.	5.8 × 10^2^
**3Mut** ^[21]^	paraoxon	65 °C	123.6 ± 12.8	71.0 ± 7.27	1.59 ± 0.114	4.5 ± 0.8 × 10^4^
	paraoxon	25 °C	8.7 ± 0.6	6.91 ± 0.47	0.380 ± 0.067	1.8 ± 0.5 × 10^4^
	m-paraoxon	65 °C	72.9 ± 9.0	43.1 ± 5.3	0.744 ± 0.22	5.8 ± 0.9 × 10^4^
	paraoxon	65 ° + 0.025% SDS	55.2 ± 3.2	31.7 ± 1.86	0.325 ± 0.05	1.0 ± 0.2 × 10^5^
	paraoxon	25 °C + 0.025% SDS	15.0 ± 0.39	8.6 ± 0.24	0.107 ± 0.012	0.8 ± 0.1 × 10^5^
**4Mut**	paraoxon	70 °C	206.4 ± 18.3	122.0 ± 10.3	0.747 ± 0.02	1.6 ± 0.2 × 10^5^
	paraoxon	25 °C	55.8 ± 1.4	33.0 ± 0.8	0.645 ± 0.053	5.1 ± 0.5 × 10^4^
	m-paraoxon	70 °C	50.9 ± 12.0	30.1 ± 7.1	0.871 ± 0.043	3.5 ± 1.0 × 10^4^
	paraoxon	70 °C +0.025% SDS	343.8 ± 13.8	203.4 ± 8.2	0.475 ± 0.051	4.3 ± 0.7 × 10^5^
	paraoxon	25 °C + 0.025% SDS	61.6 ± 1.0	36.4 ± 0.6	0.366 ± 0.017	1.0 ± 0.1 × 10^5^

**Table 3:** Kinetic analysis of *Sso*Pox3Mut and 4Mut on nerve agents.

Nerve agents	*Sso*Pox 3Mut *k*_cat_/K_M_(M^−1^ s^−1^)	*Sso*Pox 4Mut *k*_cat_/K_M_(M^−1^ s^−1^)
Tabun	6.87 × 10^1^	3.2 × 10^2^
Sarin	2.77 × 10^2^	4.2 × 10^2^
Soman	4.32 × 10^1^	4.2 × 10^2^
Cyclosarin	7.63 × 10^1^	N.A.
VX	N.A.	N.A.

**Table 4:** Data collection and refinement of *Sso*Pox 4Mut crystal structure.

Data Collection	*Sso*Pox 4Mut
Space group	*P2_1_2_1_2_1_*
Cell dimensions: a,b,c (Å)	84.138, 105.009, 153.651
Resolution (Å)	46.03-2.813
Upper resolution shell (Å)	2.913 -2.813
Measured reflections ^a^	385,386 (39,037)
Unique reflections	33,783 (3,252)
Multiplicity	11.4 (11.6)
Completeness (%)	99.07 (96.79)
Average I/ σ (I)	14.50 (2.20)
Wilson B-factor (Å^2^)	62.38
R-pim	0.0419 (0.332)
CC1/2	0.998 (0.897)
Refinement Statistics	
Reflections used in refinement	33,498 (3,252)
Reflections used for R-free	1,681 (159)
R-work	0.2178 (0.3506)
R-free	0.2498 (0.3709)
Number of non-hydrogen atoms	9424
Number of protein atoms	9412
Solvent	4
RMS(bonds) (Å)	0.011
RMS(angles) (^o^)	1.42
Ramachandran favored (%)	95.42
Ramachandran allowed (%)	4.50
Ramachandran outliers (%)	5.73
Average B-factor (Å^2^)	70.86

^a^ The values in parentheses refer to the data of the corresponding upper resolution shell.

## Supplementary Materials

Supplementary materials can be found at https://www.mdpi.com/1422-0067/21/5/1683/s1.

## Abbreviations

PLL	Phosphotriesterase-Like-Lactonase
StEP	Staggered Extension Process
OP	organophosphate
CWA	chemical warfare agent
*bd*PTE	phosphotriesterase from *Brevundimonas diminuta* MG
DrOPH/Dr0930	phosphotriesterase-like-lactonase from *Deinococcus radiodurans*
GKL/GKaP	phosphotriesterase-like-lactonase from *Geobacillus kaustophilus*
StLois	stepwise loop insertion strategy
CD	circular dichroism
AHL	acyl-homoserine lactone
TBBL	5-thiobutyl butyrolactone
C8-HSL	N-octanoyl homoserine lactone
C10-HSL	N-decanoyl homoserine lactone
ICP-MS	Inductively Coupled Plasma mass spectrometry
OpdA	phosphotriesterase from *Agrobacterium radiobacter* P230
IPTG	Isopropyl β-D-1-thiogalactopyranoside
DTNB	5,5′-Dithiobis(2-nitrobenzoic acid)
AChE	acetylcholinesterase
