# Supplementary material for: Structural and Functional Characterization of New SsoPox Variant Points to the Dimer Interface as a Driver for the Increase in Promiscuous Paraoxonase Activity
Source: Int J Mol Sci. 2020 Mar 1;21(5):1683. doi: 10.3390/ijms21051683 (PMC7084321; doi:10.3390/ijms21051683)
Supplement: Supplementary file 1 [file ijms-21-01683-s001.pdf]

Supporting Information

Structural and Functional Characterization of New *Sso*pox Variant Points to the Dimer Interface as a Driver for the Increase in Promiscuous Paraoxonase Activity

Yoko Suzumoto [1], Orly Dym [2], Giovanni N Roviello [3], Franz Worek [4], Joel L Sussman [2] and Giuseppe Manco [1,*]

[1] Institute of Biochemistry and Cell Biology, CNR, 80131 Naples, Italy
[2] The Weizmann Institute of Science, Rehovot, Israel
[3] Institute of Biostructures and Bioimages, CNR, 80131 Naples, Italy
[4] Bundeswehr Institute of Pharmacology and Toxicology, Munich, Germany
* Correspondence: Giuseppe Manco[1] Institute of Biochemistry and Cellular Biology, CNR, 80131 Naples, Italy

```
SsoPox3Mut      ATGAGAA-TA--CCA--TTAGTTGGGAAAGATTCAATAGAATCTAAGGACATAGGATTTACGCTAATTCATGAACATTTAAGAGT-TTTTAGCGAA----
SisLac          ATGAGAA-TA--CCA--TTAGTTGGGAAAGAGCCAATAGAAGCTGAGGATATGGGATTTACGCTGATTCATGAACATTTAAGAGT-TTTTAGTGAA----
SacPox          ATGACAAAAATTCCT--CTTGTAGGAAAAGGTGAAATATCACCTGGAGAAATGGGTTTTACTTTAATACATGAGCATTTAAGGGT-CTTCAGTGAA----
VmoLac          ATGGTACGCA--TCAGCATCGCTGGTGGAAACGAAATTGATCCGGGTAGTATGGGTTTAACCCTGTTTCATGAGCATTTACGCCT-GATCACCGAA----
GKL             ATGGCGGAGA--TGGTAGAAACGGTATGCGGGCCGGTGCCGGTGGAACAGCTTGGCAAAACGCTCATCCACGAGCATTTCCTCTTCGGTTATCCAGGGTT
Dr0930          ATGACGG-CA-----CA--GACGGTGACGGGCGCAGTCGCGGCGGCTCAACTCGGAGCGACGCTGCCCCACGAACACGTGATTTTCGGCTACCCCGGCTA
                ***      *             *            *              * **    **  *    ** ** **  *     *     *

SsoPox3Mut      GCGGTCAGACAACAATGGCCCCATCTATATAACGAAGATGAGGAGTTCAG-AAACGCTGTAAATGAGGTTAAAAGGGCAATGCAATTTGGAGTAAAGACT
SisLac          GCAGTCAGATATCAATGGCCTCATTTGTACAATGAAGATGAGGAGTTAAG-AAACGCTGTAAATGAGGTTAAAAGGGCTATGCAATTTGGAGTAAAGACT
SacPox          CCAGTTAGATATCAATGGCCACATCTTTATAATGAAGATGAGGAGTTAAA-AAATGCAGTAAATGAAGTAAAGACAATAATGTCATATGGTGTTAAGACC
VmoLac          GTGGTTCGCTGGAATTGGCCACATCTCTACAACGAAGATGAGGAACTCAA-ACGTGCGATCGATGCGGTCAATGCCGCGAAGAAATATGGCGTGAAAACG
GKL             TCAAGGCGATGTGACG--CGCGGCACGTTCCGTGAAGACGAGTCGCTTCG-CGTCGCAGTCGAGGCGGCGGAAAAGATGAAGCGGCACGGCATTCAAACG
Dr0930          CGCGGGCGACGTGACG--CTCGGGCCATTCGACCACGCGGCAGCGCTCGCAAGCTGCA-CCGAAACGGCGCGGGCGCTGCTGGCGCGCGGTATTCAGACG
                       *     *    *        *      * *  *      *        **     *    *             *      **  *  * **

SsoPox3Mut      ATAGTAGATCCCACTGTAA-TGGGATTGGGTAGGGACATCAGATTTATGG AAAAAGTGGTTAAGGCTACCGGGATAAATTTAGT-TGCGGGGACGGGGAT
SisLac          ATAGTGGATCCTACAGTAA-TGGGATTGGGAAGGGACATTAGATTTATGG AAAAGGTAGTTAAGACCACTGGGATAAATTTGGT-AGCAGGAACGGGGAT
SacPox          ATCGTGGATCCCACTGTCA-TGGGTTTAGGGAGAGACATTAGATTCAGTG AGAAGGTCGTGAAAGAAACAGGTATAAATGTGAT-TGCAGCAACGGGGTT
VmoLac          ATCATCGACTTGACCGTTGCTGGGATTG-GCTGTGACGTTCGCTTTAACG AGAAAGTCGCGAAAGCCACGGGTGTCAACATCAT-CATGGGTACTGGCTT
GKL             GTTGTCGATCCGACGCCGA-ACGATTGCGGGCGCAACCCGGCGTTTTTGC GGCGCGTCGCTGAAGAGACGGGGCTGAACATTATTTGCG-CCACCGGCTA
Dr0930          GTGGTGGACGCCACCCCCA-ACGACTGCGGACGCAACCCGGCCTTCCTGC GCGAGGTGAGCGAGGCAACCGGCCTCCAGATTCTGTGCG-CGACCGGCTT
                 *  * **    **        *  *   *  *  **      **           **     *    ** **  *  *  *  *        ** **

SsoPox3Mut      TTAC-ATATATATCGAC---TTACCTTTCTATTTCTTAAATAGG----------------TCAATTGATGAGATAGCTGACTTGTTTATTCATGATATA
SisLac          TTAC-ATATACGTTGAC---TTACCTTTCTATTTCTTAAATAGG----------------TCAATTGATGAGATAGCTGACCTATTTATTCACGATATA
SacPox          GTAT-ACTTACACTGAT---TTACCTTTCTTCTTCAATGGAAGA----------------TCATTGGAAGAGATTGCAGAATTATTAATACATGATATA
VmoLac          TTAC-ACGTATACCGAA---ATTCCGTTTACTTCAAGAATCGT-----------------GGGATTGACTCGTTAGTCGATGCCTTTGTACACGATATT
GKL             TTATTATGAA-GGGGAAGGGGCGCCGCCGTACTTCCAATTCCGCCGGCTTCTC-GGAACA-GCAGAAGATGACATTTACGACATGTTTATGGCCGAGCTG
Dr0930          TTATTACGAG-GGCGAGGGCGCCACGACCTACTTCAAGTTCCGC--GCTTCTCTGGGTGACGCCGAAAGCGAAATCTACGAGATGATGCGGACCGAGGTG
                 **  *        **        *    *  ***       *                               *    **     *       **  *

SsoPox3Mut      AAAGAGGGAATACAAGGTACTCTCAATAAAGCTGGCTTCGTAAAGATAGCT-GCAGATGAACCTGGGATCACAAAGGATGTGGAGAAGGTAATAAGGGCT
SisLac          AAAGAGGGAATACAGGCTACTTCCAATAAGGCTGGCTTCGTAAAGATAGCT-GCAGACGAGCCAGGCATTACTAAAGATGTGGAGAAGGTAATAAGAGCA
SacPox          AAAAAGGGAATACAAGGGACAAATAATAGAGCAGGCTTCATTAAGGTTGCA-GCAGATGAGCCAGGTATAACGAGGGATGTAGAGAGGGCAATAAGGGCA
VmoLac          ACCATTGGCATTCAAGGCACCAATACTCGCGCAGCATTCGTGAAAGCCGTA-ATCGATTCCAGCGGTCTGACGAAAGACGTGGAAATGGCAATTCGTGCA
GKL             ACCGAGGGCATTGCCGATACCGGAATCAAGGCGGTGTCATCAAGCTCGCC-TCGAGCAAAGGGCGCATCACCGAGTACGAAAAGATGTTCTTCCGCGCC
Dr0930          ACCGAGGGCATCGCCGGCACCGGCATCCGCGCCGGGGTCATCAAGCTGGCGAGCAGCCGCGA-CGCCATCACCCCCTACGAGCAACTGTTTTTCCGGGCG
                *     ** **    *  **    *     ** *   ** * **    *                   * **     * *   *   *    *  * **

SsoPox3Mut      GCTGCCATAGCAAACAAAGAGACTAAAGTACCAATAATTACCCACTCTAACGCTCACAATAACACCGGATTAGAACAGCAAAGAATATTGACTGAAGAAG
SisLac          GCAGCTATAACCCATAAGGAGGCTAAAGTACCTATAATTACGCATTCTAATGCTCACAATAACACTGGATTAGAAGAACAGAGAATATTAATGGAAGAAG
SacPox          GCTGCTATAGCTCAGAAGGAGACTAACGTACCCATAATAACACATTCAAATGCTCATAACGGGACAGGTCTTGAGCAACAAAGGATTCTAATGGAGGAGG
VmoLac          GCCGCGAAAGCGCACATCAAGACGGATGTGCCCATCATTACCCACTCTTTCGTTGGCAATAAGAGCTCTCTGGATCTGATTCGCATTTTCAAAGAAGAAG
GKL             GCCGCCCGCGCGCAAAAAGAGACGGGCGCGGTCATCATCACCCATACGCAAGAAGG-AACG--ATGGGGCCGGAACAAGCCGCCTATTTGCTTGAGCACG
Dr0930          GCGGCGCGGGTGCAGCGCGAAACCGGCGTGCCGATCATCACCCACACTCAGGAAGGCCAGC--A-GGGACCGCAGCAAGCCGAGCTGCTGACCTCGCTCG
                ** **        *     *  *    *     ** ** ** **  *    *      *    *         *              *          *

SsoPox3Mut      GTGTTGATCCAGGGAAAATATTAATAGGTCATTTAGGTGATACAGATAATATAGATTAC-ATAAAGAAGATAGCAGATAAGGGATCCTTTATTGGATTAG
SisLac          GTGTTGATCCAGGAAAGATATTGATAGGACATTTGGGGGACACTGATAATACGGATTAT-ATAAAGAAGATAGCAGATAAGGGATCCTTTATCGGATTAG
SacPox          GTGTAGACCCAGGGAGAGTGCTAATAGGTCACTTGGGGGACACTGATAACGTGGATTAC-ATAAAGAAGATAGCCGATAAAGGCTCGTTTGTAGGTCTAG
VmoLac          GGGTGGATTTGGCTCGTACCGTGATTGGCCATGTTGGCGATACAGACGACATCTCCTTC-ATTGAACAGATTCTGCGCGAAGGAGCCTTTATCGGACTTG
GKL             GCGCCGATCCGAAAAAAATTGTCATCGGCCATATGTGCGGCAACACGGACCCGGACTATCATC-GAAAGACGCTTGCTTACGGCGTTTACATTGCGTTTG
Dr0930          GCGCGGACCCGGCGCGCATCATGATCGGGCACATGGACGGCAACACCGACCCGGCCTACCACC-GCGAGACGCTGCGCCACGGCGTGAGCATCGCCTTTG
                * *  **              * ** ** **  *    *  *      *       *   *      ***         * **        * *   * *

SsoPox3Mut      ATAGATATGGTTTA---GATTTATT---CCTACCTGTTGATA---AGAGAAATGAAACGACCTTAAGACTAATCAAAGATGGTTATTCAGATAAGATAAT
SisLac          ATAGATATGGTTTA---GATTTGTT---CTTACCAGTTGATA---AGAGAAATGAAACAACATTGAAACTAATCAAGGATGGTTACTCTGATAGGATAAT
SacPox          ATAGATACGGTCTA---GATCTATT---CTTACCTATAGATA---AAAGGAACGAGGTGTTGTTGAAATTAATTAAAGATGGATACTTGGACAGGATTAT
VmoLac          ATCGCTTTGGCCTG---GACATTTA---TCTGCCGCTGGATA---AACGCGTGAAAACTGCGATTGAGCTGATCAAACGTGGTTGGATTGACCAGCTTCT
GKL             ACCGCTTCGGCATCCAAGGGATGGTCGGCGCGCCGACCGATGAGGAGCGGGTGCGGACGCTCCTTGCTCTGCTCCGCGATGGGTACGAGAAACAAATTAT
Dr0930          ACCGCATCGGCTTGCAGGGCATGGTGGGCACCCCCACCGACGCCGAGCGGCTAAGCGTGCTGACCACGCTGCTCGGCGAGGGCTACGCCGACCGGCTGCT
                *  *    **  *    *   *          **    **     *  *                    *  *       ** *      *     *  *

SsoPox3Mut      GATCTCTCACGATTATTTATGCACATTCGACGCTGG-AAC----TGCAAAACCAGAATATAAACCTAAGCTTGC-TCCAAGA-TGGAGTATAACTCTAAT
SisLac          GATTTCACATGACTATTGTTGCACAATTGACTGGGG-GAC----TGCTAGACCAGAGCTTAAACCTAAACTCGC-TCCAAGA-TGGAGTATGGCACTAAT
SacPox          GGTGTCACAAGATTACTGTTGCACAATTGACTGGGG-GAT----AGCAAAGCCGGAGTACAAACCTAAACTAGC-TCCAAAA-TGGAGTATGAGTTTAAT
VmoLac          GTTGTCGCATGACTATTGCCCGACAATTGATTGGTA-TCC----------TCCGGAAGTTGTACGTTCAACCGT-CCCGGAT-TGGACAATGACGCTGAT
GKL             GCTGTCGCATGACAC-TG-TCAACGTTTGGCTCGGTCGTCCGTTTACGCTGCCGGAACCGTTTGCGGAAATGA--TGAAAAATTGGCATGTCGAGCATTT
Dr0930          GCTCTCGCACGACAG-CATCTGGC-ACTGGCTGGGACGCCCGCCGGCCATCCCCGAAGCC--GCCTTGCCCGCCGTCAAGGACTGGCACCCTCTCCACAT
                * * ** ** **           *    *                      ** **                           ***             *

SsoPox3Mut      ATTTGAGGATACGATACCGTTCTTAAAGAGAAATGGAGTGAATGAAGAGGTTATAGCTACAATATTTAAGGAAAATCCGAAAAAGTTCTTC------AG-
SisLac          ATTTGAGGATACCATACCGTTCTTAAAGAAAAATGGAGTGAGTGAGGAGGTTATAGATATAATATTCAAGGAGAATCCGAAAAAATTCTTC------AG-
SacPox          ATTTACAGACGTTATACCCTCAATTAAGAGAGCAGGAGTAACTGATGAGCAGTTGCATGTAATCTTCGTAAAGAATCCAGCTAGACTATTT------AG-
VmoLac          CTTCGAGAAAGTGATTCCTCGCATGCGGAGTGAAGGTATTACCGAGGAACAGATTAACCGGGTTCTCATCGATAACCCACGCCGTCTGTTTACTGGCCG-
GKL             GTTTGTGAACATCATCCCCGCGCTGAAAAATGAAGGAATCCGCGACGAAGTGCTTGAGCAAATGTTCATCGGCAATCCGGCGGCGCTGTTC--TC--GGC
Dr0930          CTCCGACGACATCTTGCCTGATCTGCGGCGCCGGGGCATCACCGAGGAGCAGGTGGGGCAGATGACGGTGGGCAACCCGGCCCGGCTGTTC------GG-
                 *      *     * **     *          **  *    ** **     *        *          ** **       * **        *

SsoPox3Mut      CTAA
SisLac          CTAA
SacPox          TTAG
VmoLac          TTAA
GKL             TTGA
Dr0930          GTAA
                 *
```

Figure S1. Multiple sequence alignment of 6 homologous PLL genes utilized in this study by CLUSTAL W 1.83. Conserved nucleotides are marked by asterisk. The gene encoding *vmolac* was optimized for the expression in *E. coli* and cloned into vector pEX-A2 by Eurofins Genomics, Germany.

```
SsoPox3Mut         1    --MRIPLVGKDSIESKDIGFTLIHEHLRVFSEAVRQQWPHLY-NEDEEFRNAVNEVKRAMQFGVKTI
SisLac             1    --MRIPLVGKEPIEAEDMGFTLIHEHLRVFSEAVRYQWPHLY-NEDEELRNAVNEVKRAMQFGVKTI
SacPox             1    -MTKIPLVGKGEISPGEMGFTLIHEHLRVFSEPVRYQWPHLY-NEDEELKNAVNEVKTIMSYGVKTI
VmoLac             1    -MVRISIAGGNEIDPGSMGLTLFHEHLRLITEVVRWNWPHLY-NEDEELKRAIDAVNAAKKYGVKTI
GKL                1    MAEMVETVC-GPVPVEQLGKTLIHEHFLFGYPGFQGDVTRGTFREDESLRVAVEAAEKMKRHGIQTV
Dr0930             1    --MTAQTVT-GAVAAAQLGATLPHEHVIFGYPGYAGDVTLGPFDHAAALASCTETARALLARGIQTV
                                    .     * ** ***.                                   *..*.

SsoPox3Mut        65    VDPTVMGLGRDIRFMEKVVKATGINLVAGTGIYIYI-DLPFYFLNRS-----IDEIADLFIHDIK
SisLac            65    VDPTVMGLGRDIRFMEKVVKTTGINLVAGTGIYIYV-DLPFYFLNRS-----IDEIADLFIHDIK
SacPox            66    VDPTVMGLGRDIRFSEKVVKETGINVIAATGLYTYT-DLPFFFNGRS-----LEEIAELLIHDIK
VmoLac            66    IDLTVAGIGCDVRFNEKVAKATGVNIIMGTGFYTYTE-IPFYFKNRG-----IDSLVDAFVHDIT
GKL               67    VDPTPNDCGRNPAFLRRVAEETGLNIICATGYYYEGEGAPPYFQFRRLLGTAEDDIYDMFMAELT
Dr0930            65    VDATPNDCGRNPAFLREVSEATGLQILCATGFYYEGEGATTYFKFRASLGDAESEIYEMMRTEVT
                        .* *    * .  *   *   * .... .**              *         . .    ..

SsoPox3Mut       124    EGIQGTLNKAGFVKIAADEPGITKDVEKVIRAAAIANKETKVPIITHSNAHNNTGLEQQRILTEE
SisLac           124    EGIQATSNKAGFVKIAADEPGITKDVEKVIRAAAITHKEAKVPIITHSNAHNNTGLEEQRILMEE
SacPox           125    KGIQGTNNRAGFIKVAADEPGITRDVERAIRAAAIAQKETNVPIITHSNAHNGTGLEQQRILMEE
VmoLac           125    IGIQGTNTRAAFVKAVIDSSGLTKDVEMAIRAAAKAHIKTDVPIITHSFVGNKSSLDLIRIFKEE
GKL              132    EGIADTGIKAGVIKLASSKGRITEYEKMFFRAAARAQKETGAVIITHTQEGT-MGPEQAAYLLEH
Dr0930           130    EGIAGTGIRAGVIKLASSRDAITPYEQLFFRAAARVQRETGVPIITHTQEGQ-QGPQQAELLTSL
                         **  *  .*...*        *      .****     .   ****.      . .    .

SsoPox3Mut       189    GVDPGKILIGHLGDTDNIDYIKKIADKGSFIGLDRYGLD----LFLPVDKRNETTLRLIKDGYSD
SisLac           189    GVDPGKILIGHLGDTDNTDYIKKIADKGSFIGLDRYGLD----LFLPVDKRNETTLKLIKDGYSD
SacPox           190    GVDPGRVLIGHLGDTDNVDYIKKIADKGSFVGLDRYGLD----LFLPIDKRNEVLLKLIKDGYLD
VmoLac           190    GVDLARTVIGHVGDTDDISFIEQILREGAFIGLDRFGLD----IYLPLDKRVKTAIELIKRGWID
GKL              196    GADPKKIVIGHMCGNTDPDYHRKTLAYGVYIAFDRFGIQGMVG-APTDEERVRTLLALLRDGYEK
Dr0930           194    GADPARIMIGHMDGNTDPAYHRETLRHGVSIAFDRIGLQGMVG-TPTDAERLSVLTTLLGEGYAD
                        *    .  ***     .  .       *  ...**           .   *      *. .*

SsoPox3Mut       250    KIMISHDYLCTFD-AGTAKPEYKPKLAPRWSITLIFEDTIPFLKRNGVNEEVIATIFKENPKKFFS-
SisLac           250    RIMISHDYCCTID-WGTARPELKPKLAPRWSMALIFEDTIPFLKKNGVSEEVIDIIFKENPKKFFS-
SacPox           251    RIMVSQDYCCTID-WGIAKPEYKPKLAPKWSMSLIFTDVIPSIKRAGVTDEQLHVIFVKNPARLFS-
VmoLac           251    QLLLSHDYCPTID-WYP--PEVVRSTVPDWTMTLIFEKVIPRMRSEGITEEQINRVLIDNPRRLFTGR-
GKL              260    QIMLSHDTVNVWLGRPFTLPEPFAEMMKNWHVEHLFVNIIPALKNEGIRDEVLEQMFIGNPAALFSA-
Dr0930           258    RLLLSHDSIWHWLGRPPAIPEAALPAVKDWHPLHISDDILPDLRRRGITEEQVGQMTVGNPARLFG-
                         ...*.*                           .    .* ..  *. .*  .  .   **  .*.
```

Figure S2. Structure-based protein sequence alignment of 6 homologous PLL enzymes utilized in this study. Protein sequence alignment was performed using Swiss-pdb viewer 4.1.0, with manual improvement.

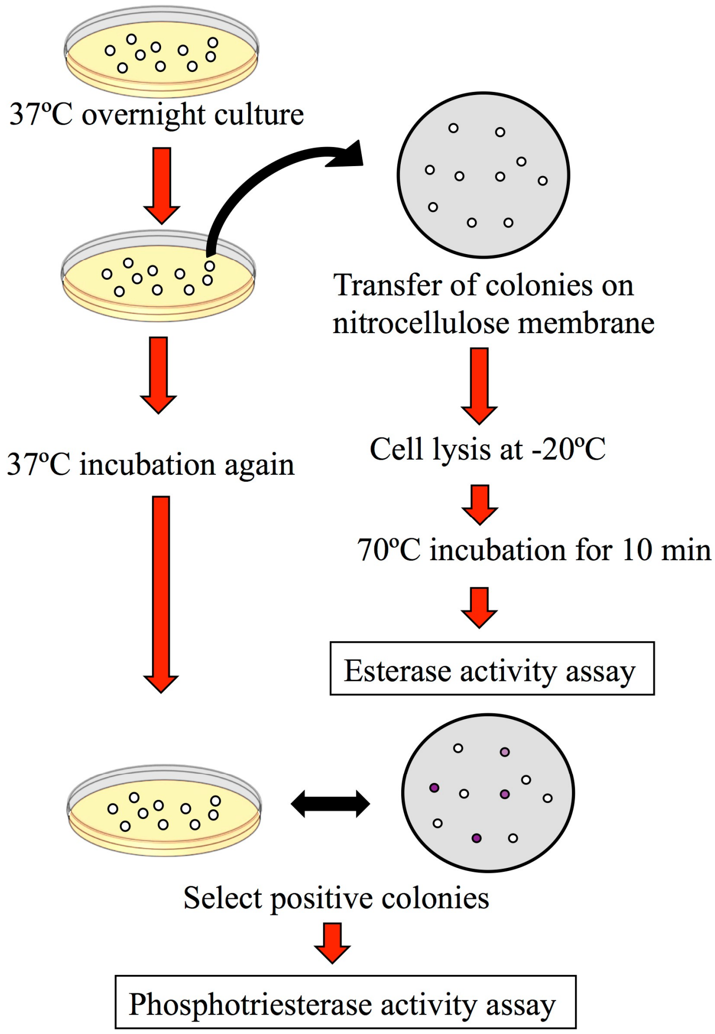

Figure S3. Scheme for the screening for PLL mutant library generated by DNA StEP using 6 homologous PLL genes. First, colonies expression mutant PLL enzymes were transferred to nitrocellulose membrane for *in situ* esterase assay. Secondly, cells were lysed by repeated incubations at −20 °C and room temperature. Esterases derived from mesophilic host were inactivated by incubation at 70 °C. Subsequently, esterase activity assay was performed by incubating nitrocellulose membrane in the reaction solution prepared by mixing 10 mg β-naphthyl acetate in 500 μl MeOH and 50 mg Fast Blue RR in 50 ml of 50 mM Tris-HCl (pH 8.5). After incubation for 10 min under gentle shaking, nitrocellulose membrane was washed with distilled water and air-dried. Significantly positive colonies were selected for secondary phosphotriesterase activity screening using paraoxon as substrate.

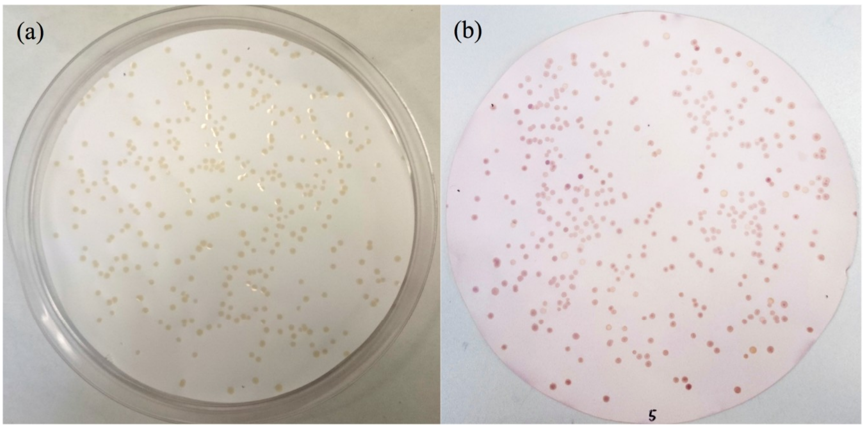

Figure S4. *In situ* esterase activity assay. (**a**) colonies transferred on nitrocellulose membrane for in situ assay. (**b**) shows nitrocellulose membrane after esterase activity assay. The intensity of violet color of each colony varies according to the esterase activity of the mutant enzyme expressed.

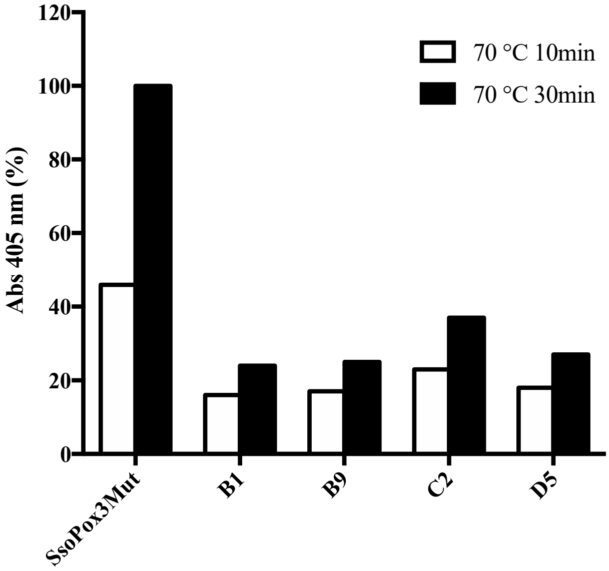

Figure S5. Comparison of paraoxonase activity with 3Mut and 4 variants. Generation of *p*-nitrophenol, the hydrolysis product of paraoxon, was detected by measuring absorbance at 405 nm. Data collection was performed at two time points (10 min and 30 min) after 70 °C incubation. The absorbance obtained at each measurement was converted in percentage by considering the value in *Sso*Pox3Mut after 30 min incubation at 70 °C as 100%.

```
B1              1     --MRIPLVGKEPIEAEDMGFTLIHEHLRVFSEAVRYQWPHLY-NEDEELRNAVNEVKRAMQFGVKTI
B9              1     --MRIPLVGKEPIEAEDMGFTLIHEHLRVFSEAVRQQWPHLY-NEDEEFRNAVNEVKRAMQFGVKTI
C2              1     --MRIPLVGKDSIESKDIGFTLIHEHLRVFSEAVRQQWPHLY-NEDEEFRNAVNEVKRAMQFGVKTI
D5              1     --MRIPLVGKEPIEAEDMGFTLIHEHLRVFSEAVRYQWPHLY-NEDEELRNAVNEVKRAMQFGVKTI
SsoPox3Mut      1     --MRIPLVGKDSIESKDIGFTLIHEHLRVFSEAVRQQWPHLY-NEDEEFRNAVNEVKRAMQFGVKTI
SisLac          1     --MRIPLVGKEPIEAEDMGFTLIHEHLRVFSEAVRYQWPHLY-NEDEELRNAVNEVKRAMQFGVKTI
SacPox          1     -MTKIPLVGKGEISPGEMGFTLIHEHLRVFSEPVRYQWPHLY-NEDEELKNAVNEVKTIMSYGVKTI
VmoLac          1     -MVRISIAGGNEIDPGSMGLTLFHEHLRLITEVVRWNWPHLY-NEDEELKRAIDAVNAAKKYGVKTI
GKL             1     MAEMVETVC-GPVPVEQLGKTLIHEHFLFGYPGFQGDVTRGTFREDESLRVAVEAAEKMKRHGIQTV
Dr0930          1     --MTAQTVT-GAVAAAQLGATLPHEHVIFGYPGYAGDVTLGPFDHAAALASCTETARALLARGIQTV
                                  .     * ** ***.                                   *..*.

B1              65    VDPTVMGLGRDIRFMEKVVKTTGINLVAGTGIYIYID-LPFYFLNRS-----IDEIADLFIHDIK
B9              65    VDPTVMGLGRDIRFMEKVVKATGINLVAGTGIYIYID-LPFYFLNRS-----IDEIADLFIHDIK
C2              65    VDPTVMGLGRDIRFMEKVVKATGINLVAGTGIYIYID-LPFYFLNRS-----IDEIADLFIHDIK
D5              65    VDPTVMGLGRDIRFMEKVVKTTGINLVAGTGIYIYVD-LPFYFLNRS-----IDEIADLFIHDIK
SsoPox3Mut      65    VDPTVMGLGRDIRFMEKVVKATGINLVAGTGIYIYI-DLPFYFLNRS-----IDEIADLFIHDIK
SisLac          65    VDPTVMGLGRDIRFMEKVVKTTGINLVAGTGIYIYV-DLPFYFLNRS-----IDEIADLFIHDIK
SacPox          66    VDPTVMGLGRDIRFSEKVVKETGINVIAATGLYTYT-DLPFFFNGRS-----LEEIAELLIHDIK
VmoLac          66    IDLTVAGIGCDVRFNEKVAKATGVNIIMGTGFYTYTE-IPFYFKNRG-----IDSLVDAFVHDIT
GKL             67    VDPTPNDCGRNPAFLRRVAEETGLNIICATGYYYEGEGAPPYFQFRRLLGTAEDDIYDMFMAELT
Dr0930          65    VDATPNDCGRNPAFLREVSEATGLQILCATGFYYEGEGATTYFKFRASLGDAESEIYEMMRTEVT
                      .* *    * .  *   *   * .... .**              *         . .    ..

B1              124   EGIQGTLNKAGFVKIAADEPGITKDVEKVIRAAAIANKETKVPIITHSNAHNNTGLEQQRILTEE
B9              124   EGIQGTLNKAGFVKIAADEPGITKDVEKVIRAAAIANKETKVPIITHSNAHNNTGLEQQRILTEE
C2              124   EGIQGTLNKAGFVKIAADEPGITKDVEKVIRAAAIANKETKVPIITHSNAHNNTGLEEQRILMEE
D5              124   EGIQATSNKAGFVKIAADEPGITKDVEKVIRAAAITHKEAKVPIITHSNAHNNTGLEEQRILMEE
SsoPox3Mut      124   EGIQGTLNKAGFVKIAADEPGITKDVEKVIRAAAIANKETKVPIITHSNAHNNTGLEQQRILTEE
SisLac          124   EGIQATSNKAGFVKIAADEPGITKDVEKVIRAAAITHKEAKVPIITHSNAHNNTGLEEQRILMEE
SacPox          125   KGIQGTNNRAGFIKVAADEPGITRDVERAIRAAAIAQKETNVPIITHSNAHNGTGLEQQRILMEE
VmoLac          125   IGIQGTNTRAAFVKAVIDSSGLTKDVEMAIRAAAKAHIKTDVPIITHSFVGNKSSLDLIRIFKEE
GKL             132   EGIADTGIKAGVIKLASSKGRITEYEKMFFRAAARAQKETGAVIITHTQEGT-MGPEQAAYLLEH
Dr0930          130   EGIAGTGIRAGVIKLASSRDAITPYEQLFFRAAARVQRETGVPIITHTQEGQ-QGPQQAELLTSL
                       **  *  .*...*        *      .****     .   ****.      . .    .

B1              189   GVDPGKILIGHLGDTDNIDYIKKIADKGSFIGLDRYGLD----LFLPVDKRNETTLKLIKDGYSD
B9              189   GVDPGKILIGHLGDTDNIDYIKKIADKGSFIGLDRYGLD----LFLPVDKRNETTLKLIKDGYSD
C2              189   GVDPGKILIGHLGDTDNTDYIKKIADKGSFIGLDRYGLD----LFLPVDKRNETTLKLIKDGYSD
D5              189   GVDPGKILIGHLGDTDNIDYIKKIADKGSFIGLDRYGLD----LFLPVDKRNETTLKLIKDGYSD
SsoPox3Mut      189   GVDPGKILIGHLGDTDNIDYIKKIADKGSFIGLDRYGLD----LFLPVDKRNETTLRLIKDGYSD
SisLac          189   GVDPGKILIGHLGDTDNTDYIKKIADKGSFIGLDRYGLD----LFLPVDKRNETTLKLIKDGYSD
SacPox          190   GVDPGRVLIGHLGDTDNVDYIKKIADKGSFVGLDRYGLD----LFLPIDKRNEVLLKLIKDGYLD
VmoLac          190   GVDLARTVIGHVGDTDDISFIEQILREGAFIGLDRFGLD----IYLPLDKRVKTAIELIKRGWID
GKL             196   GADPKKIVIGHMCGNTDPDYHRKTLAYGVYIAFDRFGIQGMVG-APTDEERVRTLLALLRDGYEK
Dr0930          194   GADPARIMIGHMDGNTDPAYHRETLRHGVSIAFDRIGLQGMVG-TPTDAERLSVLTTLLGEGYAD
                      *    .  ***     .  .       *  ...**           .   *      *. .*

B1              250   RIMISHDYCCTID-WGTARPELKPKLAPRWSMALIFEDTIPFLKKNGVSEEVIDIN----PKKFFS
B9              250   RIMISHDYCCTID-WGTARPELKPKLAPRWSMALIFEDTIPFLKKNGVSEEVIDII----PKKFFS
C2              250   RIMISHDYCCTID-WGTARPELKPKLAPRWSMALIFEDTIPFLKKNGVSEEVIDIKFIEIPKKFFS
D5              250   RIMISHDYCCTID-WGTARPELKPKLAPRWSMALIFEDTIPFLKKNGVSEEVIDINARRIPKKFFS
SsoPox3Mut      250   KIMISHDYLCTFD-AGTAKPEYKPKLAPRWSITLIFEDTIPFLKRNGVNEEVIATIFKENPKKFFS-
SisLac          250   RIMISHDYCCTID-WGTARPELKPKLAPRWSMALIFEDTIPFLKKNGVSEEVIDIIFKENPKKFFS-
SacPox          251   RIMVSQDYCCTID-WGIAKPEYKPKLAPKWSMSLIFTDVIPSIKRAGVTDEQLHVIFVKNPARLFS-
VmoLac          251   QLLLSHDYCPTID-WYP--PEVVRSTVPDWTMTLIFEKVIPRMRSEGITEEQINRVLIDNPRRLFTGR-
GKL             260   QIMLSHDTVNVWLGRPFTLPEPFAEMMKNWHVEHLFVNIIPALKNEGIRDEVLEQMFIGNPAALFSA-
Dr0930          258   RLLLSHDSIWHWLGRPPAIPEAALPAVKDWHPLHISDDILPDLRRRGITEEQVGQMTVGNPARLFG-
                       ...*.*                           .    .* ..  *. .* .  .    *  .*.
```

Figure S6. Structure-based sequence alignment between 4 variants and 6 template PLL genes. Protein sequence alignment was performed using Swiss-Pdb viewer 4.1.0, with manual improvement.

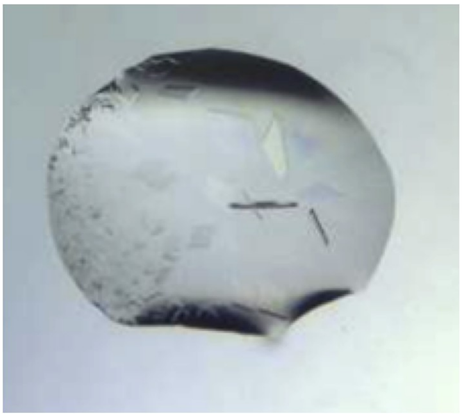

Figure S7. A representative crystal of SsoPox4Mut from which the structure was obtained.

Table S1. Sequence identities at DNA levels between 6 homologous PLL genes utilized in this study.

(%)	***Sso*Pox 3Mut**	***Sis*Lac**	***Sac*Pox**	***Vmo*Lac**	**GKL**	**Dr0930**
*Sso*Pox3Mut	100	86,8	70,7	55,7	46,7	42,6
*Sis*Lac	86,8	100	72,3	55,3	48,4	42,6
*Sac*Pox	70,7	72,3	100	55,5	47,2	44,3
*Vmo*Lac	55,7	55,3	55,5	100	47,4	47,2
GKL	46,7	48,4	47,2	47,4	100	62,9
Dr0930	42,6	42,6	44,3	47,2	62,9	100

Table S2. Sequence identities at protein levels between 6 homologous PLL enzymes utilized in this study

(%)	***Sso*Pox 3Mut**	***Sis*Lac**	***Sac*Pox**	***Vmo*Lac**	**GKL**	**Dr0930**
*Sso*Pox3Mut	100	90,5	75,2	51,9	35,8	29,9
*Sis*Lac	90,5	100	76,1	52,2	35,8	28,9
*Sac*Pox	75,2	76,1	100	52,7	35,0	32,7
*Vmo*Lac	51,9	52,2	52,7	100	31,6	31,3
GKL	35,8	35,8	35,0	31,6	100	59,1
Dr0930	29,9	28,9	32,7	31,3	59,1	100

Table S3. Sequence identities between 4 variants and template PLL genes used in DNA StEP at protein level.

**(%)**	**B1**	**B9**	**C2**	**D5**
B1	100,0	98,7	96,1	97,1
B9	98,7	100,0	97,4	96,5
C2	96,1	97,4	100,0	94,0
D5	97,1	96,5	94,0	100,0
*Sso*Pox3Mut	93,2	93,9	94,0	89,2
*Sis*Lac	97,1	95,8	94,6	98,1
*Sac*Pox	77,4	76,5	75,5	75,2
*Vmo*Lac	53,9	53,6	52,7	52,2
GKL	36,7	36,0	35,3	34,9
Dr0930	30,9	30,6	29,3	28,9
